# High-Risk Contexts for Violence Against Women: Using Latent Class Analysis to Understand Structural and Contextual Drivers of Intimate Partner Violence at the National Level

**DOI:** 10.1177/08862605221086642

**Published:** 2022-03-17

**Authors:** Laura J Brown, Hattie Lowe, Andrew Gibbs, Colette Smith, Jenevieve Mannell

**Affiliations:** 1 204274 Institute for Global Health, University College London, UK; 2 Gender and Health Research Unit, 59097 South African Medical Research Council, South Africa; 3 Centre for Rural Health, School of Nursing and Public Health, 56394 University of KwaZulu-Natal, South Africa

**Keywords:** Intimate partner violence, ecological analysis, latent class analysis, structural drivers, risk factors, colonisation, gender inequality

## Abstract

**Introduction**: Intimate partner violence (IPV) affects 1 in 3 women and poses a major human rights threat and public health burden, yet there is great variation in risk globally. Whilst individual risk factors are well-studied, less research has focussed on the structural and contextual drivers of IPV and how these co-occur to create contexts of high risk. **Methods**: We compiled IPV drivers from freely-accessible global country-level data sources and combined gender inequality, natural disasters, conflict, colonialism, socioeconomic development and inequality, homicide and social discrimination in a latent class analysis, and identified underlying ‘risk contexts’ based on fit statistics and theoretical plausibility (N=5,732 country-years; 190 countries). We used multinomial regression to compare risk contexts according to: proportion of population with disability, HIV/AIDS, refugee status, and mental health disorders; proportion of men with drug use disorders; men’s alcohol consumption; and population median age (N=1,654-5,725 country-years). Finally, we compared prevalence of physical and/or sexual IPV experienced by women in the past 12 months across risk contexts (N=3,175 country-years). **Results**: Three distinct risk contexts were identified: 1) non-patriarchal egalitarian, low rates of homicide; 2) patriarchal post-colonial, high rates of homicide; 3) patriarchal post-colonial conflict and disaster-affected. Compared to non-patriarchal egalitarian contexts, patriarchal post-colonial contexts had a younger age distribution and a higher prevalence of drug use disorders, but a lower prevalence of mental health disorders and a smaller refugee population. IPV risk was highest in the two patriarchal post-colonial contexts and associated with country income classification. **Conclusions**: Whilst our findings support the importance of gender norms in shaping women’s risk of experiencing IPV, they also point towards an association with a history of colonialism. To effectively address IPV for women in high prevalence contexts, structural interventions and policies are needed that address not only gender norms, but also broader structural inequalities arising from colonialism.

## Introduction

Violence against women (VAW) is a major human rights violation and global public health concern (WHO, 2021). The most common form of VAW is intimate partner violence (IPV), and global estimates suggest one in four women will experience physical and/or sexual violence by a husband or male intimate partner in their lifetime, and one in 10 in the past 12 months (WHO, 2021).

Despite its ubiquitous nature, there is great variation globally in past year IPV prevalence, and little understanding of why this may be the case. Regional-level variation is evident, with for instance, 4–7% of women in Europe reporting past year experience of IPV, compared to 20% in Sub-Saharan Africa and 30% in Melanesia (WHO, 2021). At the individual level, studies highlight high levels of risk factors such as young age, low socioeconomic status and alcohol consumption (and many others) in high IPV prevalence settings (Abramsky et al., 2011; Yakubovich et al., 2018). However, beyond the individual level, our understanding of contextual and structural drivers of high IPV prevalence has been undermined by a lack of analysis at an area level and a lack of a global perspective (Beyer et al., 2015; Mannell et al., 2022; Montesanti, 2015; VanderEnde et al., 2012).

Despite these gaps, the importance of structural drivers of IPV is well recognised, including community-level risks (VanderEnde et al., 2012) and contexts of poverty (Gibbs, Dunkle, et al., 2020). Feminist epidemiologists working on VAW argue that IPV is driven by gender inequalities (Heise & Kotsadam, 2015; Jewkes & Morrell, 2018), while feminist economists have argued that IPV is often driven by women’s lack of household bargaining power and their disempowerment vis-à-vis men (Hughes et al., 2015; Kabeer, 1997). Others have focused on conflict-affected settings (Ellsberg et al., 2020; Gibbs, Abdelatif, Said, et al., 2020; M. Hossain et al., 2014) or countries with high levels of neighbourhood violence (Kiss et al., 2015; Piscitelli & Doherty, 2019; Raghavan et al., 2006) to understand how these specific contexts relate to high prevalences of IPV. Intersecting systems of power (e.g. race, class, etc.) and oppression (e.g. prejudice and social discrimination) create complex social contexts which further shape women’s risk of violence (Sokoloff & Dupont, 2005). This is exemplified by the inequitable burdens imposed by climate change and natural disasters (Camey et al., 2020; Thurston et al., 2021) and colonisation (Burnette & Renner, 2017; Lugones, 2007; Mama, 2017) (e.g. on some countries, by others; on indigenous, impoverished and other minoritised communities; and on women more so than men).

The mechanisms through which these different structural and contextual drivers influence IPV are complex. For example, armed conflict has been identified as a key structural, although likely indirect, driver of IPV (Devakumar et al., 2021; Gibbs, Dunkle, et al., 2020). IPV remains one of the most common forms of violence in conflict-affected/displaced population settings (Ellsberg et al., 2020; Stark & Ager, 2011). The stress related to conflict, such as forced displacement or loss of financial stability, may be a trigger for IPV or may exacerbate ongoing violence (Wirtz et al., 2014). Environmental threats, such as natural disasters, are another indirect driver of IPV, and amplify gender inequalities and power imbalances in communities and households coping with resource scarcity and societal stress (Camey et al., 2020). The increased economic stress within households, and having to rebuild homes after property damage and loss of assets, can result in relationship stress and conflict, thereby leading to an increased risk of IPV (Epstein et al., 2020; Thurston et al., 2021). Colonialism may have increased IPV through its historical oppression and imposed patriarchal beliefs that have resulted in the devaluing of women (Lugones, 2007; Mama, 2017). Colonial rule can influence the gender system in many ways, such as by shifting norms and changing laws (Elvy, 2014). One such mechanism of influence is through military subjugation and institutionalisation into hierarchical and patriarchal structures, with, for example, men militarised, and women excluded from colonial employment opportunities (Mama, 2017).

Despite consensus that there are macro-level influences on women’s lives and their chances of experiencing IPV, there is limited understanding of how these different drivers may overlap and reinforce one another, and in what combinations, to form contexts of high IPV risk. The default position has therefore been to categorise and study IPV prevalence using simplistic divisions of high income countries (HICs) versus low and middle income countries (LMICs) (Mercy et al., 2017; Stöckl et al., 2013; WHO, 2021), or to solely focus on IPV prevalence in just one or the other (Coll et al., 2020; Peterman et al., 2015). This has not only limited our understanding of the interrelations of structural and contextual drivers of IPV, but has also depicted LMICs as having particularly high levels of IPV, further reifying notions of LMICs as inherently problematic and needing intervention (Cornwall, 2007; Escobar, 2011)

Socioeconomic development is likely associated with many drivers of IPV and focussing only on country income group classification omits valuable structural and contextual information that may be contributing to elevated IPV risk. In other words, we need to unpack the drivers that are associated with socioeconomic development and how they may in turn influence women’s experiences of violence. For example, many LMICs have a history of being colonised; colonial rule can impact a country’s economic development (in different ways, see Acemoglu, Daron and Robinson, 2017), whilst at the same time, and as discussed above, reshape gender norms in ways which may increase violence against women (Lugones, 2007; Mama, 2017). Similarly, the bulk of natural disasters occur in LMICs (CRED, 2015). This is in part due to their natural geographical vulnerability, but also to extractive and unsustainable economic development activities (S. Hossain et al., 2017). This vulnerability is further compounded by HICs’ historical disregard for the environmental consequences of their own pursuit of economic development (Faber, 2008). Environmental injustice is most strongly felt among women in LMICs, who are disproportionately negatively impacted by environmental threats and climate change; women who are already in marginalised positions are particularly vulnerable, especially those who are impoverished, indigenous, or who live in rural areas (Langer et al., 2015). Environmental threats manifest in a range of negative health outcomes for women, and as discussed above, the economic strain associated with natural disasters results in increased IPV (Camey et al., 2020).

Our paper builds on from new learnings in the field of IPV research (Camey et al., 2020; Gibbs, Dunkle, et al., 2020; Heise & Kotsadam, 2015; Kiss et al., 2015), and contributes to theoretical debates around which structural and contextual drivers are important in creating contexts that increase IPV risk. We aim to explore global differences in prevalence rates of IPV from an evidence-based perspective. Specifically, we seek to understand which of these drivers tend to co-occur and are in turn associated with higher IPV prevalence. We draw on global data to conduct an ecological country-level latent class analysis to answer the following research questions: 1) how do structural and contextual drivers of IPV pattern together to form distinct risk contexts?; and 2) how does the prevalence of women’s recent experience of physical and/or sexual IPV vary across these different risk contexts?

To answer these questions, we integrate Gibbs et al.’s framework of structural drivers (2020) and Heise and Kotsadam’s gender-focussed ecological analysis (2015) with broader contextual drivers of IPV to understand how these may cluster together. We hypothesised that variation in IPV prevalence is not simply explained by country income classification, but rather that IPV is higher in contexts with greater co-occurrences of contextual and structural drivers.

## Methods

### Derivation of IPV drivers and risk factors dataset

We conducted a review of macro-level data to identify variables suitable for use as IPV drivers and risk factors in our analyses. Although the two terms are synonymous and most often just reflect differences in qualitative and quantitative discourses, we have deliberately used the terms ‘driver’ and ‘risk factor’ throughout to contrast contextual/structural (driver) versus individual (risk factor) characteristics and to differentiate between the two steps of our analysis (detailed below). For use in our latent class analysis, we searched for data that might capture the structural drivers used in Gibbs et al.’s framework (2020) and Heise and Kotsadam’s ecological analysis (2015) (i.e. conflict, socioeconomic development, socioeconomic inequality, normalisation and acceptability of violence and gender inequality) as well as the broader contextual drivers of climate change, natural disasters and colonialism. We also looked for data related to social discrimination as we considered this another important structural driver to include. To help interpret our latent classes, we searched for data that might capture individual risk factors, such as childhood experience of violence, migration experience, disability, poor mental health, alcohol and substance use, HIV/AIDS and age. We only included freely-accessible data from rigorous and reputable sources, such as those from governmental and international organisations like the WHO, World Bank, United Nations, and academic research institutions. We imposed no restrictions on the calendar years studied and looked for data sources that covered both HICs and LMICs. Given possible secular changes in contextual and structural drivers and risk factors, we focussed on country-year level data (and data that could be transformed into this format) to be able to account for this variation over time in our analyses. We compared the advantages and disadvantages of different indicators in terms of how well they captured the drivers and risk factors of interest, and further limited our final selection to those with relatively good geographical and time coverage.

Our data search resulted in eight variables selected/derived to be used in our latent class analysis, six that capture structural drivers (armed conflict in past 25yrs, socioeconomic development [GDP], socioeconomic inequality [Gini Index], normalisation and acceptability of violence [homicide rate per 100,000], the Gender Inequality Index, social discrimination [high proportion of population not wanting neighbours from minority groups]) and two that capture contextual drivers (severe natural disaster in past 5yrs, and ever colonised). We used proportion of refugees, disability prevalence, prevalence of mental health disorders, alcohol consumption among men and prevalence of substance use disorders among men, HIV/AIDS prevalence among 15–49 year olds, and population median age to capture additional risk factors. As these additional risk factors were also measured at the macro-level, they may more accurately capture structural or contextual, rather than individual, influences on IPV. However, as these variables relate more to conceptualisations of individual-level risk factors for IPV (Heise, 1998; e.g., Yakubovich et al., 2018), and because latent class analysis becomes both illogical and unfeasible with too many indicator variables, we include them in later stages of analysis (detailed below), rather than in the latent class analysis itself. More information on the selected/derived drivers and risk factors is provided in Supplement Appendix A.

IPV drivers and risk factors were combined from the different data sources, matching on country-year using ISO alpha-3 codes. Given that country-level indicators are unlikely to change drastically from year to year over the short term (Heise & Kotsadam, 2015), we used a last observation carried forward approach to impute missing data. Data were carried forward for up to a maximum of 10 years for most variables, although for some countries the maximum time span reached 12 years for homicide rate, 22 years for Gini and 30 years for disability prevalence. Further, we excluded country-year records for which more than 50% of the drivers and risk factors had missing information (after imputation; *N* = 10,278 records). This resulted in an IPV drivers and risk factors dataset comprised of 5,732 country-years from 190 countries, with a median of 31 (range 3–31) years of data included per country.

### IPV estimates

In order to investigate the association between risk contexts and IPV prevalence, we reviewed available IPV prevalence estimates and compiled a dataset of estimates for past 12 months physical and/or sexual violence by a current or former intimate partner (identified by our search strategy as detailed in Supplement Appendix B). To increase comparability, we only included estimates that combined physical and sexual violence (thereby capturing women who experienced *only* physical violence, *only* sexual violence, or *both* physical and sexual violence) and excluded estimates that referred to just one of these, or another type of violence. We restricted our dataset to women’s self-reports as men tend to under-report their perpetration to a greater extent than women under-report their experience (Chan, 2011). Where reports covered a timespan of more than 1 year, we assigned the end year of a survey, for example, Haiti’s 2015–2016 Demographic Health Survey (DHS) was assigned the year 2016, and the United Nations Multi-Country study of Men and Violence (UNMCS) surveys were assigned 2013 as the report indicated they took place between 2010 and 2013 (Fulu et al., 2013). Where prevalence estimates for multiple age spans were provided, we chose estimates for the widest age spans available, which in most cases was 15–49 years, although this included 18–49 or 18–50 years in some cases. We used sub-national estimates on 17 occasions where national estimates were not available (see Supplement Appendix B). This resulted in a dataset of past 12 months physical and/or sexual IPV experience prevalence estimates comprising 371 country-years, reflecting 163 different countries and a timespan covering 1993 to 2019.

For 360 of these 371 observations, IPV estimates could be matched by country-year to the IPV risk drivers and risk factors dataset. To increase the number of matched country-years and analytical sample size, we then used a last observation carried forward/backward approach to impute missing IPV data, restricting to plus or minus 5 years, with priority given to estimates obtained in earlier years. This resulted in a total sample size of 3,175 country-years to test the association between risk contexts and IPV.

We ranked the prevalence estimates (post-imputation) into quintiles of low to high IPV prevalence based on the data’s distribution. This was to help mitigate against some of the likely error in IPV estimates associated with underreporting, varying methodological approaches (Ellsberg et al., 2001; Ellsberg & Heise, 2005), and our imputation of missing data.

### Statistical analysis

Latent class analysis (LCA) creates a categorical latent variable to capture the possibility that different profiles arise because there are underlying subgroups with distinct combinations of features (Hallquist & Wright, 2014). LCA is used to derive groups based on patterns of shared characteristics that distinguish members of one group from those of another (Golder et al., 2012). In IPV research, this approach has been used at the individual level to explore links between different masculinities and IPV perpetration among men (e.g. Gibbs, Abdelatif, Washington, et al., 2020; Jewkes et al., 2020; Jewkes & Morrell, 2018), and to understand which subgroups of women experiencing IPV are at greatest risk of other problems, such as substance use and poor mental health (e.g. Golder et al., 2012). Here we used this approach to categorise country-years included in the drivers and risk factors dataset into subgroups, henceforth referred to as ‘risk contexts’. These risk contexts were chosen by considering how the eight structural and contextual IPV drivers (described above) patterned together in the dataset. These risk contexts can therefore be considered to represent underlying constructs. This approach was chosen because LCA goes beyond variable-centred approaches to reveal something meaningful about underlying subgroups (i.e. co-occurrences of risk) (Bogat et al., 2005; Golder et al., 2012). Furthermore, LCA can help to address methodological challenges that arise in subgroup analysis, including a high Type I error rate and low statistical power (Lanza & Rhoades, 2013).

We accounted for clustering at the country level by including a clustered sandwich estimator of the variance-covariance matrix, and included calendar year as a model covariate. Models were estimated with 20 Expectation-Maximisation iterations and 200 draws of random starting values to ensure that a global rather than a local (sub-optimal) solution was found. Parameters were freely estimated (i.e. means and variances were not constrained to be equal across latent classes) and we allowed for correlations between continuous indicators in each class due to our theoretical predictions that indicator variables would be associated with each other (Ng, 2019). We used a combination of model fit statistics and class separation measures to aid with model selection. Specifically, we examined model fit with the AIC, BIC, sample size adjusted BIC, and the Lo-Mendell-Rubin Likelihood Ratio Test comparing k to k-1 classes (Lo et al., 2001; Ng, 2018)^,^ and we compared neatness of classification with normalised entropy (Ng & Schechter, 2017), Average Posterior Probability and Odds of Correct Classification (Nagin, 2005). To further assist with model selection, the two, three, four and five-class model response profiles were additionally evaluated for substantive meaning. Analyses were conducted in Stata/MP 16.1 (StataCorp, n.d.). Example syntax is included in Supplement Appendix D.

We descriptively explored the association between risk context and country income group classification. We then described the prevalence (or mean value, as appropriate) of the additional risk factors (proportion of refugees, disability prevalence, prevalence of mental health disorders, alcohol consumption among men, substance use disorder prevalence among men, HIV/AIDS prevalence among 15–49 year olds, and population median age) in the identified risk contexts. These analyses were weighted by the posterior probabilities of class membership, to account for the uncertainty in assigning risk contexts. To further account for the effect of calendar time and the relationships between risk factors on these comparisons, we also conducted unadjusted and mutually-adjusted multinomial regression analyses controlling for calendar year. We calculated predicted marginal probabilities of risk context membership associated with different combinations of these risk factors by setting their levels to very low (at 10^th^ percentile of distribution), low (25^th^ percentile), mid (50^th^ percentile [median]), high (75^th^ percentile) and very high (90^th^ percentile) levels (except for median age which was reverse-coded so that very low was 90^th^ percentile and very high 10^th^ percentile etc.) and seeing which risk contexts these were most likely to map to.

Finally, we investigated whether the identified risk contexts were associated with prevalence of IPV. We first compared the proportion of country-years in each IPV prevalence quintile across risk contexts using chi-squared tests, accounting for clustering at the country level. To additionally take account of secular trends, we used logistic regression controlling for calendar year to assess how risk context membership was associated with the probability of having a high IPV prevalence (in the top quintile)^1^. All analyses were again weighted by posterior probabilities of risk context membership. To assess the usefulness of the HIC versus LMIC split in defining risk contexts for IPV, we ran three models: Model 1 with just risk context as a predictor of IPV, Model 2 with just income classification as a predictor of IPV, and Model three containing both variables.

### Sensitivity analysis

We repeated our analyses restricted to non-imputed data (*N* = 1,451 country-years for LCA analyses; *N* = 134 for IPV analyses) to check whether substantive findings changed.

## Results

### Sample description

As shown in Supplement Appendix A Table A1, in our combined dataset (denominators vary; max *N* = 5732 country-years), 60% of country-years experienced armed conflict in the last 25 years, and 34% experienced severe natural disaster(s) in the last 5 years. 75% had been colonised and 25% had high levels of social discrimination. The median gender and socioeconomic inequality levels were 0.43 (i.e. medium inequality; IQR 0.24–0.57) and 0.39 (i.e. relatively reasonable income gap; IQR 0.33–0.45), respectively. The median GDP was $42,400 million (IQR $10,800-$230,000 million). The median homicide rate was 3.3 per 100,000 (IQR 1.4–8.8).

In terms of the additional risk factors explored, in the overall sample, refugees comprised a median of 0.1% of the population (IQR 0.0–0.4%). The median disability prevalence was 1.9% (IQR 1.3–4.1%), mental health disorders 12.3% (IQR 11.2–14.7%), substance use disorders among men 0.9% (IQR 0.7–1.2%) and HIV/AIDS among 15–49 yr olds 0.1% (IQR 0.0–0.8%). The median amount of alcohol consumed annually by men was 9 litres (IQR 3.6–14.9 L), and the median age of the overall sample was 24 years (IQR 18.6–33.1 years).

### Missingness

LCA indicator (i.e. contextual and structural driver) missingness ranged from 0-64% and additional risk factor missingness ranged from 0.1% to 71% (Table A1 in Supplement Appendix A). Missingness was not completely at random. Some countries and years were better represented in the analytical sample than others. For example, whilst the majority of countries had 31 country-years, Hong Kong SAR, China only had 7 (2014–2020) and Virgin Islands only had 3 (2018–2020) (Table A3 in Supplement Appendix C).

### Latent class analysis

**Figure 1.**
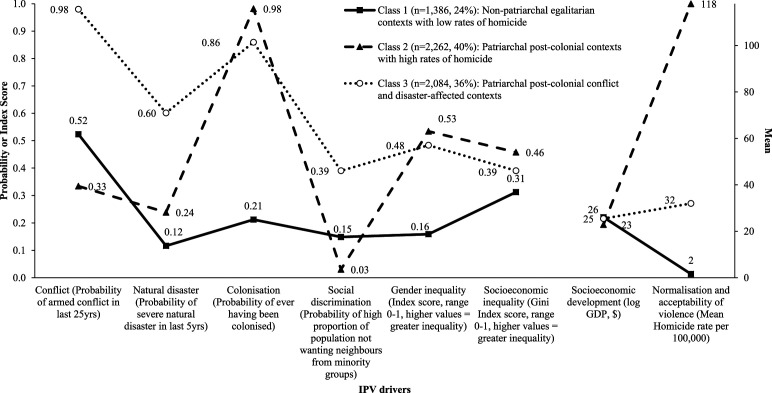
Levels of IPV drivers in each risk context

Model fit indices and class separation measures suggested three- and four-class models as candidate solutions (see Table A4 in Supplement Appendix E). Our review of the response profiles confirmed that the three-class model had the strongest theoretical basis (Figure 1). **Class 1 - Non-patriarchal egalitarian contexts with low rates of homicide** (*N* = 1,386, 24% of country-years) – was defined by a generally low level of IPV drivers. Most notably, country-years in this class tended to have high socioeconomic development along with low levels of gender inequality and socioeconomic inequality. They also had low probabilities of ever being colonised and experiencing recent severe natural disasters, as well as low homicide rates. They had middling probabilities of armed conflict and social discrimination. **Class 2 - Patriarchal post-colonial contexts with high rates of homicide** (*N* = 2,262, 40% of country-years) – was defined by relatively high levels of most of the IPV drivers. Country-years in this class tended to have low socioeconomic development along with high levels of gender inequality and socioeconomic inequality. They also had a high probability of ever being colonised, as well as high homicide rates. They had, however, low probabilities of armed conflict and social discrimination, and a mid-level probability of recent severe natural disasters. **Class 3 – Patriarchal post-colonial conflict and disaster-affected contexts** (*N* = 2,084, 36% of country-years) – was also defined by relatively high levels of several IPV drivers. Country-years in this class had high probabilities of armed conflict, recent severe natural disasters and social discrimination. However, they had a mid-level probability of ever being colonised and mid-levels of socioeconomic development, gender inequality and socioeconomic inequality.

The association between risk context and country income group classification was statistically significant, *X*^*2*^ (189, *N* = 5,732 country-years) = 2,686.13, *p <0.001*. Only 27.8% of country-years in non-patriarchal egalitarian contexts (Class 1) were classified as LMICs (Low 3.1%, Lower-middle 5.1%, Upper-middle 19.6%), whilst 92.3% and 93.1% were LMICs in the two patriarchal post-colonial contexts (Class 2: Low 33.5%, Lower-middle 36.2%, Upper-middle 22.6%; Class 3: Low 32.4%, Lower-middle 38.1%, Upper-middle 22.6%).

Table 1 (left-hand side) presents descriptive associations between risk contexts and additional risk factors for IPV. For example, the mean percentage (95% CI) of refugees in Class 1 country-years was 0.4% (0.2%, 0.5%), but 0.9% for both Class 2 (0.2%, 1.6%) and Class 3 (0.2%, 1.6%). The identified non-patriarchal egalitarian contexts with low rates of homicide (Class 1) had the lowest prevalence of HIV/AIDS and the oldest median age, but also the highest prevalence of mental health disorders and substance use disorders and the highest levels of alcohol consumption. The patriarchal post-colonial contexts with high rates of homicide (Class 2) had the highest prevalence of HIV/AIDS and the lowest median age but also the lowest prevalence of mental health disorders and substance use disorders. The patriarchal post-colonial conflict and disaster-affected contexts (Class 3) had the lowest levels of alcohol consumption.^2^ These results were confirmed in the formal regression analysis accounting for time-based variation (Table 1, right side). After adjustment, country-years in Class 2 had on average an 8% higher prevalence of substance use disorders among men compared to those in Class 1 (95% CI 0.2%, 15.9%), as well as a median age of 0.7 years younger (−1.1yrs, −0.3yrs). Country-years in Class 2 did however also have lower prevalences of refugees and mental health disorders compared to those in Class 1. On average, compared to those in Class 1, country-years in Class 3 had higher prevalences of substance use disorders among men and a lower median age, but also lower prevalences of disability, refugees and mental health disorders.

**Table 1.** Associations between Risk Contexts and Additional IPV Risk Factors.

	Descriptive Associations between Risk Contexts and Additional IPV Risk Factors						Multinomial Regression of Additional IPV Risk Factors (Reference: Class 1: Non-Patriarchal Egalitarian Contexts with Low Rates of Homicide)							
	*n*						Class 2: Patriarchal Post-colonial Contexts with High Rates of Homicide vs Class 1				Class 3: Patriarchal Post-colonial Conflict and Disaster-affected Contexts vs Class 1			
		Total	Class 1: Non-patriarchal Egalitarian Contexts with Low Rates of Homicide	Class 2: Patriarchal Post-colonial Contexts with High Rates of Homicide	Class 3: Patriarchal Post-colonial Conflict and Disaster-affected Contexts	*p*-value	Unadjusted	*p*-value	Mutually Adjusted (*N* = 1,234)	*p*-value	Unadjusted	*p*-value	Mutually Adjusted (*N* = 1,234)	*p*-value
		Mean (95% CI)	Mean (95% CI)	Mean (95% CI)	Mean (95% CI)		Est. (95% CI)		Est. (95% CI)		Est. (95% CI)		Est. (95% CI)	
Refugees (% Pop.)	4,755	0.8 (0.3, 1.3)	0.4 (0.2, 0.5)	0.9 (0.2, 1.6)	0.9 (0.2, 1.6)	0.309	0.1 (0.0, 0.2)	0.092	−0.4 (−0.7, 0.0)	**0.033**	0.1 (0.0, 0.2)	0.098	−0.5 (−0.9, −0.2)	**0.005**
Living with a disability (% Pop.)	1,654	3.2 (2.5, 4.0)	4.2 (1.5, 6.9)	3.8 (2.4, 5.3)	2.7 (1.9, 3.5)	0.287	0.0 (−0.2, 0.2)	0.880	−0.1 (−0.3, 0.1)	0.191	−0.2 (−0.4, 0.0)	0.121	−0.3 (−0.6, −0.1)	**0.002**
Mental health disorders (% Pop.)	5,725	13.0 (12.6, 13.3)	14.5 (13.8, 15.2)	12.1 (11.8, 12.5)	12.9 (12.4, 13.4)	**<0.001**	−0.5 (−0.6, −0.3)	**<0.001**	−0.7 (−1.4, −0.1)	**0.029**	−0.3 (−0.5, −0.1)	**<0.001**	−0.7 (−1.4, −0.1)	**0.034**
Alcohol consumption (l per capita: Men)	3,767	9.7 (8.7, 10.7)	15.5 (14.0, 16.9)	8.1 (7.0, 9.2)	7.8 (6.4, 9.1)	**<0.001**	−0.2 (−0.2, −0.1)	**<0.001**	−0.2 (−0.7, 0.2)	0.376	−0.2 (−0.3, −0.1)	**<0.001**	−0.2 (−0.6, 0.3)	0.421
Substance use disorders (% Men)	5,725	1.1 (1.0, 1.2)	1.3 (1.2, 1.5)	0.9 (0.8, 1.0)	1.2 (1.0, 1.4)	**<0.001**	−1.2 (−1.8, −0.6)	**<0.001**	8.0 (0.2, 15.9)	**0.046**	−0.3 (−0.7, 0.2)	0.246	11.7 (3.8, 19.5)	**0.004**
HIV/AIDS (% Pop. 15−49 yrs)	5,725	1.4 (0.9, 1.9)	0.2 (0.1, 0.3)	2.5 (1.3, 3.6)	1.1 (0.6, 1.5)	**<0.001**	1.6 (0.3, 2.9)	**0.014**	0.2 (−6.9, 7.4)	0.949	1.5 (0.2, 2.8)	**0.023**	0.1 (−7.1, 7.2)	0.984
Median age of total population (yrs)	5,709	26.1 (25.0, 27.2)	36.5 (35.3, 37.6)	21.9 (20.8, 22.9)	24.2 (22.9, 25.4)	**<0.001**	−0.3 (−0.4, −0.3)	**<0.001**	−0.7 (−1.1, −0.3)	**<0.001**	−0.3 (−0.4, −0.2)	**<0.001**	−0.9 (−1.3, −0.5)	**<0.001**

Est = Estimate; CI = Confidence Interval. Risk factors measured at country-year level. Descriptive associations adjusted for clustering within countries and weighted by the posterior probabilities of latent class membership. *p*-values for Wald tests for comparison of means. Multinomial models additionally adjusted for time-based variation. Unadjusted models contain one risk factor only and the number of observations (n) varies between models due to differing levels of missing data. Mutually adjusted models contain all seven risk factors together (*N* = 1,234 country-years).

**Figure 2.**
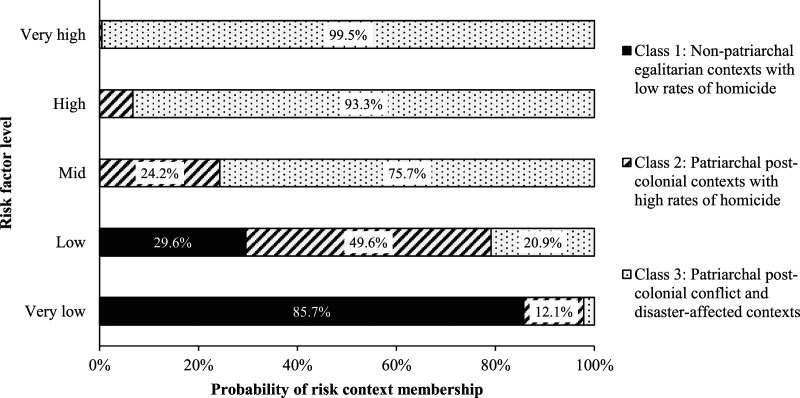
Predicted probabilities of risk context membership associated with different levels of additional IPV risk factors

Figure 2 shows marginal predicted probabilities of belonging to each risk context, assuming a range of scenarios for the additional IPV risk factors. Imagine a hypothetical country in a particular year which had very low prevalences of all of these risk factors. For this country-year, it is predicted that there is an 86% probability of being in Class 1, a 12% probability of being in Class 2, and just a 2% probability of being in Class 3 (Figure 2). In contrast, for a hypothetical country in a specific year with very high prevalences of all of these risk factors, there is almost a 100% probability that this country would belong to Class 3. Thus, Class 1 is most associated with the most favourable risk profile, and Class 3 with the worst risk factor profile.

Descriptive analyses showed that IPV prevalence differed across the three risk contexts (Table 2; *p*<0.001). Overall, IPV prevalence was lowest in non-patriarchal egalitarian contexts with low rates of homicide (Class 1) and highest in patriarchal post-colonial contexts with high rates of homicide (Class 2) and patriarchal post-colonial conflict and disaster-affected contexts (Class 3). Only 0.8% of country-years in Class 1 reported very high prevalence of IPV (i.e. 23% or more of women experiencing physical and/or sexual violence from a current/former partner in the past 12 months), whereas 29% and 25% of country-years fell in this top quintile for Classes 2 and 3, respectively.

**Table 2.** Descriptive Association between Risk Contexts and IPV Prevalence.

	Total		Class 1: Non-patriarchal Egalitarian with Low Rates of Homicide		Class 2: Patriarchal Post-colonial with High Rates of Homicide		Class 3: Patriarchal Post-colonial Conflict and Disaster-affected	
	*n*	% (95% CI)	*n*	% (95% CI)	*n*	% (95% CI)	*n*	% (95% CI)
Quintiles of physical and/or sexual IPV prevalence								
1, Low (1–4.9%)	644	20.3 (14.7, 27.4)	471	52.5 (38.6, 65.9)	40	4.5 (1.3, 14.4)	133	10.3 (5.1, 19.8)
2 (5–8%)	699	22.0 (16.6, 28.5)	340	37.5 (26.0, 50.7)	193	21.2 (12.3, 34.1)	166	12.0 (7.4, 19.0)
3 (8.5–13.7%)	571	18.0 (13.2, 24.1)	83	9.2 (4.0, 19.4)	146	14.7 (8.1, 25.4)	342	26.5 (18.4, 36.7)
4 (13.8–22.4%)	631	19.9 (14.6, 26.5)	1	0.1 (0.0, 0.6)	282	30.1 (19.3, 43.8)	348	26.2 (18.2, 36.1)
5, High (22.9–64.1%)	630	19.8 (14.3, 26.9)	10	0.8 (0.2, 2.8)	270	29.4 (18.7, 43.1)	350	25.0 (16.5, 36.0)

Adjusted for clustering within countries and weighted by the posterior probabilities of latent class membership. *X^2^* (154, *N* = 3,175 country-years) = 1410.80, *p* **< 0.001.**

The logistic regression models (Table 3) showed that the patriarchal post-colonial contexts were much more likely to have a high prevalence of IPV than the non-patriarchal contexts (Model 1: Class 2 OR 50.6, 95% CI 13.2–194.1; Class 3 OR 45.2, 95% CI 12.2–167.0). Similarly high odds of high IPV were found for income group classification, with lower middle (OR 48.4, 95% CI 6.1–384.8) and low income country-years (OR 144.2, 95% CI 18.4–1132.0) far more likely to have a high IPV prevalence than high income country-years (Model 2). When both variables were included in the same model, these effect sizes were considerably attenuated. After controlling for income group, there was no longer an association between risk contexts and IPV prevalence in the highest quintile, although confidence intervals were wide. Conversely, after accounting for risk context, income group was still strongly associated with high IPV prevalence, although differences only persisted between low income and high income countries (OR 39.6, 95% CI 1.8–852.1).

**Table 3.** Logistic Regression of High Prevalence of IPV on Risk Context and Income Group.

	Model 1: Risk Context		Model 2: Income Group		Model 3: Risk Context and Income Group	
	OR (95% CI)	*p*-value	OR (95% CI)	*p*-value	OR (95% CI)	*p*-value
Risk context						
1: Non-patriarchal egalitarian with low rates of homicide	ref.	**<0.001**			ref.	0.270
2: Patriarchal post-colonial with high rates of homicide	50.6 (13.2, 194.1)				6.0 (0.7, 53.1)	
3: Patriarchal post-colonial conflict and disaster-affected	45.2 (12.2, 167.0)				5.1 (0.6, 45.8)	
Income group						
High			ref.	**<0.001**	ref.	**0.001**
Upper middle			9.2 (1.3, 66.1)		3.3 (0.2, 45.6)	
Lower middle			48.4 (6.1, 384.8)		13.3 (0.7, 266.5)	
Low			144.2 (18.4, 1132.0)		39.6 (1.8, 852.1)	

*N* = 3,175 country-years. Adjusted for clustering within countries and time-based variation and weighted by the posterior probabilities of latent class membership. High prevalence of IPV: fifth quintile of physical and/or sexual IPV prevalence, OR: odds ratio of having a high prevalence of IPV. CI: Confidence Interval. *p*-values for Wald tests for overall effects of variables.

### Sensitivity analysis

The sensitivity analysis considering a complete case analysis (i.e. without imputation) showed very similar results. The LCA profiles found a three-class solution remained the best model. The associations with the additional risk factors remained similarly patterned, although we could no longer include disability due to high levels of missingness. In the multinomial analysis of risk factors, adjusted models showed largely consistent associations, although HIV/AIDS prevalence was higher in Classes 2 and 3 than in Class 1, and refugee population and substance use disorders were no longer associated at the 5% level with risk context, although levels remained higher in Classes 2 and 3 than in Class 1. IPV prevalence showed similar between-class differences, with 0% of Class 1 country-years in the two highest IPV quintiles, and 33% and 16% of country-years in the top quintile for Classes 2 and 3, respectively, *X*^*2*^ (80, *N* = 134) = 37.06, *p <0.001.*

## Discussion

We have used a quantitative empirical approach that captures more country-years than other cross-national comparisons (e.g. Heise & Kotsadam, 2015) to contribute to understandings of macro-level drivers of intimate partner violence. We set out to go beyond dichotomising countries into HICs and LMICs, to assess how contextual and structural drivers of violence co-occur. We hypothesised that there are likely to be distinct clusters of drivers that correlate with heightened IPV risk at the macro-level, and that whilst these drivers will be socioeconomically patterned to some extent, a country’s income level is not the only driving force behind violence prevalence.

Our ecological latent class analysis identified three distinct risk contexts: 1) non-patriarchal egalitarian with low rates of homicide; 2) patriarchal post-colonial with high rates of homicide, and 3) patriarchal post-colonial conflict and disaster-affected, which were in turn associated with different prevalences of IPV (low in Class 1, but high in Classes 2 and 3). These distinctions highlight the importance of gender inequality and colonial history in driving high rates of IPV. Our findings therefore lend support to feminist theories which centre the importance of patriarchal norms and women’s disempowerment in contributing to IPV risk (Yodanis, 2004), whilst at the same time highlight that patriarchal norms and colonialism are closely tied. In addition, the different combinations of the other contextual and structural drivers and their interrelations in Classes 2 and 3 also speak to heterogeneity in contexts with high IPV.

As highlighted in the introduction, colonialism is an important contextual driver of IPV because of how it has imposed patriarchal beliefs that devalue women (Lugones, 2007; Mama, 2017). In addition, the historical oppression of colonialism is argued to have resulted in intergenerational trauma and continued structural inequities (Burnette & Renner, 2017). These can manifest as poverty and discrimination and have knock-on effects for mental health, alcoholism and substance use among men and women alike (Jones, 2008); again further exacerbating women’s vulnerability to violence (Greene et al., 2021). IPV perpetration may be an expression of internalised oppression resulting from historical trauma among both men and women, but the intersecting oppressions of colonialism, racism and sexism experienced by indigenous women mean that they still disproportionately experience IPV and suffer its negative impacts compared to indigenous men (Burnette & Figley, 2017; Burnette & Renner, 2017; Jones, 2008). In our study we found that mental health disorders, alcohol consumption and substance use disorders were *lower* in Classes 2 and 3. We did however see some of the other additional negative legacies in the distribution of drivers across the three risk contexts, such as the high levels of armed conflict in Class 3 and the lower GDP and higher socioeconomic inequality in both of the patriarchal post-colonial contexts. The importance of colonialism in contributing to high rates of IPV is hugely under-acknowledged, despite anthropologists and social scientists discussing this link for years (Elvy, 2014; Montesanti, 2015). Our study provides one of the first pieces of quantitative evidence that highlights the central role that colonialism has played in driving increases in IPV.

Although comparable levels of gender inequality and colonisation history were present, homicide rates were much higher in Class 2 (118 per 100,000) than in Class 3 (32 per 100,000). Normative violent behaviour may increase IPV risk through a culture of violence that encourages aggression both outside and inside the home (Kiss et al., 2015). Whilst other studies have indexed this with subjective attitudinal measures (VanderEnde et al., 2012), we instead used the objective measure of homicide rate due to its higher level of geographical coverage (attitudes to wife-beating are only available via the DHS and so restricted to LMICs). Given that gang involvement accounts for a large share of homicides (UNODC, 2019), there may be important links with IPV worth exploring. Women’s involvement increases their exposure to gang-related violence, but many women are additionally subjected to violence from their intimate partners who are also part of these networks (Ahlenback & Clugston, 2020; Bourgois, 2002). Our paper focused on IPV prevalence, but it is likely that IPV severity may also increase for women involved in gangs, with for example, the use of firearms resulting in greater chances of serious injuries and death. Men’s possession of firearms may mean that relationship power dynamics are tipped in their favour; guns facilitate coercion and intimidation and may therefore increase the likelihood of IPV perpetration (Ahlenback & Clugston, 2020). In addition, the relationship between gang involvement and IPV is likely bi-directional, with IPV victims being more likely to get involved with gangs, sometimes seeking affiliation as a means of protection from violent partners (Shaw & Skywalker, 2017).

The intentional homicide measure used in this analysis excludes deaths resulting from armed conflict. Even though these numbers are often far less than deaths as a result of gang involvement (UNODC, 2019), the impact of armed conflict is devastating, not least for women. Studies have suggested that armed conflict increases IPV by ingraining patriarchal social relationships, normalising violence, entrenching poverty, increasing alcohol and substance use, and worsening mental health (Gibbs, Abdelatif, Said, et al., 2020; Gibbs, Dunkle, et al., 2020; Kelly et al., 2018; Saile et al., 2013). Our analysis lends some support to these links, with, for example, the conflict-affected contexts (Class 3) also having high levels of gender inequality, a low GDP, and a relatively high prevalence of substance use and mental health disorders. These IPV drivers and risk factors have also been shown to increase when natural disasters occur (Rezaeian, 2013; Thurston et al., 2021). Therefore, it is not surprising that armed conflict and natural disasters clustered together in our study, given these well-established interlinkages (Camey et al., 2020). As described in our introduction, both often result in a loss of property and assets, which increases household stress, and in turn increases IPV risk (Epstein et al., 2020; Thurston et al., 2021; Wirtz et al., 2014). Of note, whilst our measure for natural disasters from the EM-DAT database included biological disasters, COVID-19 was not listed for any of the included country-years at the point of extraction (early February 2021). However, the increased levels of intimate partner violence experienced during the pandemic by many women globally (Sánchez et al., 2020; UN Women & Women Count, 2021) suggests further support for our findings that disasters are important contextual drivers. Both natural disasters and conflict may result in displacement. Displaced populations face heightened violence, for example, in acquiring natural resources, and the physical layout and social characteristics of refugee camps further increase VAW (Ager et al., 2018; Kwiringira et al., 2018). Furthermore, there appears to be a bi-directional relationship between environmental threats and armed conflict. On the one hand, climate change and environmental threats can increase the risk of state fragility and fuel social unrest, potentially leading to violent conflict (Camey et al., 2020; Rüttinger, 2017). On the other hand, conflict-affected and fractured state-society relations also increase vulnerability to climate change and disasters by depleting assets that could be used in adaptation and mitigation efforts, further contributing to environmental degradation (Camey et al., 2020; Rüttinger, 2017). This bi-directional relationship also highlights how countries affected by conflict and/or disasters are likely to be resource-constrained and therefore classified as LMICs.

Whilst a country’s income classification is likely to be linked with several of the macro-level IPV drivers we have studied here, differences in IPV levels are unlikely directly attributable to income levels alone. This is supported by a review of community-level studies that found that standard of living was not consistently related to IPV risk (VanderEnde et al., 2012). Although the association between income and IPV remained after adjustment for risk context in our study, socioeconomic development has previously been suggested to be indirectly related to IPV risk, rather than being a key driver in and of itself. Heise and Kotsadam performed a macro-level analysis in which they contested the notion that population-level differentials in violence can be explained purely by variation in socioeconomic development (2015); they found that GDP no longer predicted IPV prevalence once gender norms were accounted for. They suggest that socioeconomic development indicators are unlikely to be causally related to IPV risk, but are instead markers for more complex social processes and gender-related transformations that occur alongside economic growth. In addition, rather than our three risk contexts mapping on to high, middle and low income countries, we found that Class 1 was predominantly comprised of HICs, and Classes 2 and 3 predominantly LMICs. The identification of two separate LMIC clusters suggests that within LMICs there remain further distinct and different clusters of drivers. This suggests that categorisation by income only may miss key determinants of IPV; despite some similarities (i.e. in colonial history and gender inequality), LMICs are heterogenous, and should not be considered as a single context. Moreover, LMICs still made up more than a quarter of the country-years in Class 1– further suggesting that low IPV prevalence is not just a de facto benefit of high-income status.

After using structural and contextual drivers of IPV to divide the world into three risk contexts, we did not find that the additional risk factors we explored patterned with consistently higher levels in the two contexts with higher IPV prevalence (i.e. Classes 2 and 3). This is perhaps surprising given that HIV status, disability status, refugee status, age, substance use and mental health have been shown to be important risk factors for IPV experience and/or perpetration (Abramsky et al., 2011; Campbell et al., 2008; Capaldi et al., 2012; Greene et al., 2021; Gupta et al., 2018; Iudici et al., 2019; Keygnaert et al., 2012; Machisa et al., 2016; Peterman et al., 2015; Scheer et al., 2020; Yakubovich et al., 2018). In addition, these risk factors often co-occur to synergistically increase IPV risk (Hatcher et al., 2019; Jewkes & Morrell, 2018; Russell et al., 2013). However, this may also reflect that these risk factors were measured at the country-year, rather than individual, level. When we considered hypothetical examples with different risk factor levels (Figure 2), we were however able to recreate their syndemic relationship to some extent. Although hypothetical at the macro-level, these situations that vary from very low to very high risk may be lived realities at smaller geographical scales – for some countries, and for some couples – and serve to highlight the multi-factorial and intersectional nature of IPV risk (Scheer et al., 2020).

### Limitations

Our study has several limitations. Ideally, we would have included several other drivers and risk factors, but we were restricted by limited data availability and coverage. Relatedly, we imputed some data; we believe this is however reasonable as national-level indicators change slowly (Heise & Kotsadam, 2015) (and our sensitivity analyses without imputed data did not substantively alter our findings). Whilst we focused on country-years rather than countries as the unit of analysis to account for changes in risk contexts over time, our analysis was still cross-sectional in nature, and as such we cannot make any claims in relation to causality (Dutton, 1994). Although an ecological macro-level analysis is well-suited to exploring structural and contextual drivers, some of our included measures may have been more informatively measured at lower levels. In particular, we used country-year level estimates of IPV, which masks that IPV is experienced by individual women, not a country as a whole. In addition, in comparing our three risk contexts we examined levels of other risk factors which are more readily conceptualised as individual, rather than structural/contextual. Furthermore, we must also heed the ecological fallacy, and not infer individual-level relationships from macro-level findings (Dutton, 1994; Zeoli et al., 2019). Relatedly, our analysis obscures variation within countries. Several of our drivers and risk factors are likely to vary across smaller geospatial scales – for example, in urban versus rural communities (Beyer et al., 2015); even gender inequality, a key delineator of the different risk contexts in our analyses, is likely to vary between communities in the same country (VanderEnde et al., 2012). The absence of observed links between different risks factors at the country level may be obscuring links occurring for specific sub-populations (e.g. indigenous communities). Currently, the available global data on violence against women focuses almost exclusively on women’s experience of violence in intimate partnerships with men, and does not account for women who may be experiencing violence in same sex relationships. Our paper’s novel insights notwithstanding, future analyses would benefit from multi-level modelling which combines drivers and risk factors measured at different levels, to more accurately capture the multi-layered influences on IPV. Structural equation modelling frameworks could also be exploited to examine their causal and indirect pathways (VanderEnde et al., 2012).

## Conclusion

By considering the possibility that contextual and structural drivers of IPV pattern into latent risk contexts, we have been able to show that settings are not merely differentiated by level of risk, but rather that different drivers cluster together to create three distinct risk contexts. Although they differed in conflict, natural disasters and homicide, the two contexts which had high chances of IPV were both post-colonial and patriarchal. Whilst the observed strong links with country income suggest that comparing LMICs versus HICs provide some insight into drivers of IPV, our analyses also lend support to the idea that socioeconomic development is a marker for more complex social processes and gender-related transformations. Our findings support the importance of gender norms in shaping women’s risk of experiencing IPV (Scheer et al., 2020), but they also suggest a negative legacy of colonialism, and highlight that LMICs are heterogenous contexts with different clusters of risk. Furthermore, our analyses show that a focus on income classification obscures nuance and the heterogeneity of different contexts. Our paper has helped to unpack this nuance by demonstrating differential clustering of contextual and structural drivers in HICs versus LMICs. Interventions to reduce violence against women need to address several societal issues simultaneously and take into account the interplay of structural and contextual drivers in different contexts in order to be effective (Greenhalgh & Papoutsi, 2018; Moore et al., 2019). Violence *within* communities can only be addressed when the violence directed *against* communities (e.g. structural forms of violence including racism, extractive industries etc.) is also tackled (Sokoloff & Dupont, 2005). Key examples of IPV interventions trying to address contextual drivers and structural forms of violence include syndemic models of IPV prevention (Gibbs et al., 2014; González-Guarda et al., 2011), interventions targeting historical trauma and racism as a driver of IPV perpetration (Taft et al., 2021), as well as vital work on indigenous approaches to violence prevention (Varcoe et al., 2017). In addition, a decolonising approach to VAW research and intervention development in LMICs (Mannell et al., 2021) may be particularly helpful in finding localised solutions to the global problem of IPV.

## Supplemental Material

Supplemental Material – High-Risk Contexts for Violence Against Women: Using Latent Class Analysis to Understand Structural and Contextual Drivers of Intimate Partner Violence at the National Level Click here for additional data file. Supplemental Material for High-Risk Contexts for Violence Against Women: Using Latent Class Analysis to Understand Structural and Contextual Drivers of Intimate Partner Violence at the National Level by Laura J Brown, Hattie Lowe, Andrew Gibbs, Colette Smith and Jenevieve Mannell in J Interpers Violence

## Notes

1. We used this logistic regression approach as ordered logistic regression likelihood ratio and Brant tests suggested that the proportional odds assumption was violated (Williams, 2006).

2. We also compared medians using the Kruskall Wallis test, but unlike the Wald tests these did not take account of clustering at country-level or weighting by posterior probabilities. The results showed very similar patterns, with the only differences being that all tests of differences across classes were significant and Class 1 had the highest median prevalence of refugees.
