# Supplementary material for: High-Risk Contexts for Violence Against Women: Using Latent Class Analysis to Understand Structural and Contextual Drivers of Intimate Partner Violence at the National Level
Source: J Interpers Violence. 2022 Mar 17;38(1-2):NP1007–39. doi: 10.1177/08862605221086642 (PMC9709538; doi:10.1177/08862605221086642)
Supplement: Supplemental Material – High-Risk Contexts for Violence Against Women: Using Latent Class Analysis to Understand Structural and Contextual Drivers of Intimate Partner Violence at the National Level [file sj-pdf-1-jiv-10.1177_08862605221086642.pdf]

Appendices

Appendix A. Table A1: IPV drivers and risk factors - data sources and summaries

	Indicator<br><br>*Variable type*	Data source	Timespan - original (sample)	Geographical coverage - original (sample)	Data/indicator description	Recoding for analysis	n	Missing -ness: n (%)	Median (IQR) / n (%)
Latent class indicators: structural and contextual drivers measured at the country-year level									
**Armed conflict**	Conflict in last 25 years<br><br>*Binary (1 "Yes", 0 "No")*	Heidelberg Institute for International Conflict Research. (2020). *Conflict Barometer 2019*. HIIK. https://hiik.de/data-and-maps/datasets/?lang=en	1825-2018 (1990-2020)	243 (190) countries	The HIIK dataset is based on information gathered in its CONTRA database. Data includes substate, intrastate, interstate and trans-state conflicts detailing start year and listed by conflict	With the 2019 dataset, we used start year along with conflict descriptions to ascertain active conflict status in a given country-year. Next, the 2019 codebook was checked for conflicts closed or marked inactive in 2017, 2018 and 2019, and cross-referenced with the 2016 dataset (as this has start year information) for any additional countries. This data was used to code whether country-years were currently in active conflict and we derived whether conflict had occurred in the last 25 years.	5,732	0 (0.0%)	3,445 (60.1%)
**Social discrimination**	High social discrimination<br><br>*Binary (1 "Yes", 0 "No")*	Inglehart, R., Haerpfer, C., Moreno, A., Welzel, C., Kizilova, K., Diez-Medrano, J., Lagos, M., Norris, P., Ponarin, E., & Puranen, B. (2020). *World Values Survey: All Rounds - Country-Pooled Datafile.* JD Systems Institute & WVSA. http://www.worldvaluessurvey.org/WVSDocumentationWVL.jsp	1981-2020 (1990-2020)	104 (102) countries	Nationally representative global survey of human beliefs and values, which contains the question "On this list are various groups of people. Could you please indicate any that you would not like to have as neighbors?" – with various groups listed as options	As data was only available at the individual level, we calculated the proportion of the surveyed population (for each country-year) who mentioned the following groups: immigrants/foreign workers, people of a different race, people of a different religion, people who speak a different language (using individual sample non-response weights in these calculations). We then took the highest of these values as representing the highest level of disclosed discriminatory preferences. We then split this into quartiles and defined a binary variable separating those in the top quartile from the other three to create high vs low social discrimination categories,	2,083	3,649 (63.7%)	516 (24.8%)

						respectively.			
**Gender inequality**	Gender Inequality Index<br><br>*Continuous (bounded 0-1*)	United Nations Development Programme. (2020). *Gender Inequality Index (GII).* Human Development Reports. http://hdr.undp.org/en/indicators/68606#a	1995-2019 (1995-2020)	162 (162) countries	Composite measure reflecting inequality in achievement between women and men; comprises five indicators (adolescent fertility, maternal mortality, educational attainment, political representation, and labour force participation) over three domains of human development (reproductive health, empowerment, and the labour market). The higher the index value, the greater the disparities between women and men. Ranges from 0 (the lowest inequality between women and men) to 1 (the highest inequality) and can be classified into high (0.60 or higher), medium (0.4 to <0.6), and low (< 0.4) inequality.	Reshaped data from wide to long format.	3,762	1,970 (34.4%)	0.43 (0.24 – 0.57)
**Socioeconomic inequality**	Gini Index<br><br>*Continuous (bounded 0-100*)	World Bank. (2020). *Gini index (World Bank estimate).* Development Research Group. https://data.worldbank.org/indicator/SI.POV.GINI	1967-2018 (1990-2020)	165 (163) countries	Data are based on primary household survey data obtained from government statistical agencies and World Bank country departments. The index measures extent to which the distribution of income (or, in some cases, consumption expenditure) among individuals or households within an economy deviates from a perfectly equal distribution. A Lorenz curve plots the cumulative percentages of total income received against the cumulative number of recipients, starting with the poorest individual or household. The Gini index measures the area between the Lorenz curve and a hypothetical line of absolute equality, expressed as a proportion of the maximum area under the line. A Gini index of 0 represents perfect equality, while an index of 1 implies perfect inequality.	Reshaped data from wide to long format.	3,935	1,797 (31.4%)	0.39 (0.33 – 0.45)
**Socioeconomic development**	GDP, PPP (current international $)	World Bank. (2020). *GDP, PPP (current international $).* International Comparison Program, World Development	1990-2019 (1990-2020)	197 (184) countries	This indicator provides values for gross domestic product (GDP) expressed in current international dollars, converted by purchasing power parity (PPP) conversion	Reshaped data from wide to long format. Log transformed due to skewed distribution.	5,471	261 (4.6%)	Log transformed: 24.5 (23.1 – 26.2)

	Continuous (log transformed)	Indicators database, Eurostat-OECD PPP Programme. https://data.worldbank.org/indicator/NY.GDP.MKTP.PP.CD			factor.				Original: $42,400m ($10,800m - $230,000m)
**Normalisation and acceptability of violence**	Homicide rate per 100,000<br><br>*Continuous (log transformed)*	DATAUNODC. (2020). *Homicide rate.* United Nations Office on Drugs and Crime. https://dataunodc.un.org/content/data/homicide/homicide-rate	1990-2018 (1990-2020)	203 (169) countries	Annual United Nations Crime Trends Survey (UN-CTS) with additional data sourced from the most reliable sources available. Intentional homicide = Unlawful death inflicted upon a person with the intent to cause death or serious injury. Inclusions: Murder; honour killing; serious assault leading to death; death as a result of terrorist activities; dowry-related killings; femicide; infanticide; voluntary manslaughter; extrajudicial killings; killings caused by excessive use of force by law enforcement/state officials. Exclusions: Death due to legal interventions; justifiable homicide in self-defence; attempted intentional homicide; homicide without the element of intent is non-intentional homicide; non-negligent or involuntary manslaughter; assisting suicide or instigating suicide; illegal feticide; euthanasia	Log transformed due to skewed distribution.	4,260	1,472 (25.7%)	Log transformed : 1.2 (0.3 – 2.2) Original: 3.3 (1.4 – 8.8)
**Climate change and natural disasters**	Severe natural disaster in last 5yrs<br><br>*Binary (1 "Yes", 0 "No")*	Centre for Research on the Epidemiology of Disasters (CRED). (2020). *Emergency Events Database (EM-DAT).* Université catholique de Louvain (UCLouvain), Belgium. https://www.emdat.be/	1900-2020 (1990-2020)	243 (190) countries	Data includes natural, technological and complex emergency events where: Deaths: 10 or more people deaths; Affected: 100 or more people affected/injured/homeless; Declaration/international appeal: Declaration by the country of a state of emergency and/or an appeal for international assistance.	We included only natural disasters (i.e. geophysical, meteorological, hydrological, biological, climatological, and extra-terrestrial). Total deaths and total affected summed across all natural disasters in a given country-year and added together to get total impact. Country-years in the top impact quintile were considered to have experienced a severe natural disaster. So as to capture longer term impacts of natural disaster exposure, we created a variable that indicated whether there has been a severe (top quintile) natural disaster in the last 5 years.	5,732	0 (0.0%)	1,940 (33.9%)
**Colonisation**	Ever	Becker, B. (2019). *Colonial*	1462-1984	243 (190)	The Colonial Dates Dataset	The COLDAT colonies and start	5,732	0	4,309

	colonised<br><br>*Binary (1 "Yes", 0 "No")*	*Dates Dataset (COLDAT), V2*. Harvard Dataverse. https://doi.org/10.7910/DVN/T9SDEW	(1990-2020)	countries	(COLDAT) aggregates information on the reach and duration of European colonial empires from renowned secondary sources.	and end dates of colonial rule were used to derive colonisation history (country-years which preceded start of colonisation were coded as No). We supplemented the COLDAT data to identify whether the country was a post-soviet country (i.e. had been colonised by Russia) or had been colonised by the USA. In addition, we manually searched colonial histories for countries not included in the COLDAT list so that more information could be added to our dataset (we note that this information may be less reliable).		(0.0%)	(75.2%)
Risk factors for further analysis: individual risk factors measured at the country-year level									
**Migration experience**	Refugee population (% Pop.)<br><br>*Continuous (bounded 0-100*)	World Bank. (2020). *Refugee population by country or territory of asylum.* United Nations High Commissioner for Refugees (UNHCR) and UNRWA through UNHCR's Refugee Data Finder at unhcr.org/refugee-statistics. https://data.worldbank.org/indicator/SM.POP.REFG	1960-2019 (1990-2020)	164 (164) countries	Refugee population by country or territory of asylum = the number people who have crossed an international border to find sanctuary and have been granted refugee or refugee-like status or temporary protection.	Reshaped into long format. Divided by total population and multiplied by 100 to create % variable, taking account of country size.	4,755	977 (17.1%)	0.1 (0.0 - 0.4)
**Disability**	Living with a disability (% Pop.)<br><br>*Continuous (bounded 0-100*)	International Minnesota Population Centre. (2020). *Integrated Public Use Microdata Series, International: Version 7.3.* IPUMS. https://doi.org/https://doi.org/10.18128/D020.V7.3	1800s-2017 (1990-2020)	72 (72) countries	"Disabled" indicates whether the person reported a disability of any kind (IPUMS harmonised variable across different surveys and questions from various population-based surveys)	Only used data from 1900 onwards. Individual survey data used to generate country-year level proportions of population with a disability taking into account sample person weights. Proportions multiplied by 100 to give percentage prevalence estimates.	1,654	4,078 (71.1%)	1.9 (1.3 - 4.1)
**Poor mental health**	Mental health disorders (% Pop.)<br><br>*Continuous (bounded 0-100*)	Institute for Health Metrics and Evaluation (IHME). (2020). *GBD Results Tool: B.6 Mental disorders.* http://ghdx.healthdata.org/gbd-results-tool	1990-2019 (1990-2020)	204 (189) countries	B.6 Mental disorders includes schizophrenia, depressive disorders, eating disorders, autism, ADHD, conduct disorders, idiopathic developmental intellectual disability, other mental disorders. GBD data is based on a combination of sources, including medical and national records, epidemiological data, in addition to	None.	5,725	7 (0.1%)	12.3 (11.2 - 14.7)

					survey data. Where raw data for a particular country is scarce, epidemiological data and meta-regression models are used based on available data from neighbouring countries. Data therefore present an estimate (rather than official diagnosis) of mental health.				
**Alcohol**	Alcohol consumption (l per capita: Men)<br><br>*Continuous*	World Bank. (2020). *Total alcohol consumption per capita, male (liters of pure alcohol, projected estimates, male 15+ years of age)*. World Health Organization, Global Health Observatory Data Repository (apps.who.int/ghodata). https://data.worldbank.org/indicator/SH.ALC.PCAP.MA.LI	2000-2018 (2000-2020)	187 (183) countries	Defined as the total (sum of recorded and unrecorded alcohol) amount of alcohol consumed per male (15 years of age or older) over a calendar year, in litres of pure alcohol, adjusted for tourist consumption.	Reshaped into long format.	3,767	1,965 (34.3%)	9.0 (3.6 - 14.9)
**Substance use**	Substance use disorders (% Men)<br><br>*Continuous (bounded 0-100)*	Our World in Data. (2020). *Prevalence of drug use disorders, males vs females, 2017*. Institute for Health Metrics and Evaluation (IHME), Global Burden of Disease. https://ourworldindata.org/grapher/prevalence-of-drug-use-disorders-males-vs-females	1990-2017 (1990-2020)	195 (189) countries	The share of men with a substance use disorder.	None.	5,725	7 (0.1%)	0.9 (0.7 - 1.2)
**HIV/AIDS**	HIV/AIDS (% Pop. 15-49 yrs)<br><br>*Continuous (bounded 0-100)*	Our World in Data. (2020). *HIV/AIDS: Prevalence in the total population.* Institute for Health Metrics and Evaluation (IHME), Global Burden of Disease. https://ourworldindata.org/hiv-aids	1990-2017 (1990-2020)	195 (189) countries	The share of people aged 15 to 49 years old who are infected with HIV	None.	5,725	7 (0.1%)	0.1 (0.0 - 0.8)
**Age**	Median age of total population (yrs)	United Nations Department of Economic and Social Affairs. (2019). *SA1/POP/5: Median age by*	1950-2020 (1990-2020)	201 (188) countries	Estimates and Medium variant, 1950 - 2100.	Reshaped from wide to long format. We only used estimates to 2020, not future projections.	5,709	23 (0.4%)	24.0 (18.6 - 33.1)

	Continuous	*aggregate and constituents, 1950-2100 (years).* Population Dynamics - World Population Prospects. https://population.un.org/wpp/Download/Standard/Population/

Data summaries refer to fully compiled, partially imputed, and missingness-restricted dataset (i.e. our analytical sample) where N = 5,732 country-years. Percentages are adjusted for clustering at country-level but don't account for time-based variation (medians don't account for either). See Appendix F for data acknowledgements.

Appendix B. Table A2: IPV estimates (women's reports of experiencing physical and/or sexual IPV in the last 12m) and data sources – arranged alphabetically and chronologically by ISO code

Country-year	Denom. (yrs)	Est. (%)	Included in UN SDG database ?	Source	Link	Other source: detail	Sub-national estimate?
**AFG 2015**	15-49	46.1	Yes	DHS	https://unstats.un.org/sdgs/indicators/database/		No
**AFG 2018**	15-49	35	No	WHO VAW 2018	https://www.who.int/publications/i/item/9789240022256		No
**AGO 2016**	15-49	25.9	Yes	DHS	https://unstats.un.org/sdgs/indicators/database/		No
**AGO 2018**	15-49	25	No	WHO VAW 2018	https://www.who.int/publications/i/item/9789240022256		No
**ALB 2018**	15-49	6	No	WHO VAW 2018	https://www.who.int/publications/i/item/9789240022256		No
**ARG 2015**	18-69	2.7	No	Other (via UN Women Global Database on Violence against Women)	https://evaw-global-database.unwomen.org/en	Ministerio de Justicia y Derechos Humanos de la Nación. 2017. Primer Estudio Nacional sobre Violencias contra la Mujer 2015, Basado en la International Violence Against Women Survey (IVAWS), 1a ed. Ministerio de Justicia. Ciudad Autónoma de Buenos Aires: Edicициones SAIJ.	No
**ARG 2018**	15-49	4	No	WHO VAW 2018	https://www.who.int/publications/i/item/9789240022256		No
**ARM 2016**	15-49	3.5	Yes	DHS	https://unstats.un.org/sdgs/indicators/database/		No
**ARM 2018**	15-49	5	No	WHO VAW 2018	https://www.who.int/publications/i/item/9789240022256		No
**AUS 2003**	16+	4	No	IVAWS (via UN Women matrix)	https://www.who.int/publications/i/item/9789240022256		No
**AUS 2016**	18+	2.2	No	Other (via UN Women Global Database on Violence against Women)	https://evaw-global-database.unwomen.org/en	Australian Bureau of Statistics, 2017. Personal Safety Survey 2016. Canberra, Australia.	No
**AUS 2018**	15-49	3	No	WHO VAW 2018	https://www.who.int/publications/i/item/9789240022256		No
**AUT 2012**	18-74	4	Yes	calculated by FRA based on available country data from the 2012 FRA Violence against women: an EU-wide Survey	https://unstats.un.org/sdgs/indicators/database/		No
**AUT 2018**	15-49	4	No	WHO VAW 2018	https://www.who.int/publications/i/item/9789240022256		No
**AZE 2006**	15-49	10.2	Yes	DHS (corrected UN SDG value from 9.8 to 10.2 based on what is reported in DHS report and UN Women matrix)	https://unstats.un.org/sdgs/indicators/database/		No
**AZE 2018**	15-49	5	No	WHO VAW 2018	https://www.who.int/publications/i/item/9789240022256		No
**BDI 2017**	15-49	27.8	No	DHS (corrected UN SDG value from 27.9 to 27.8 based on what is reported in DHS report)	https://dhsprogram.com/		No
**BDI 2018**	15-49	22	No	WHO VAW 2018	https://www.who.int/publications/i/item/9789240022256		No
**BEL 2012**	18-74	8	Yes	calculated by FRA	https://unstats.un.org/sdgs/indicat		No

				based on available country data from the 2012 FRA Violence against women: an EU-wide Survey	ors/database/		
**BEL 2018**	15-49	5	No	WHO VAW 2018	https://www.who.int/publications/i/item/9789240022256		No
**BEN 2018**	15-49	13.9	No	DHS	https://dhsprogram.com/		No
**BFA 2011**	15-49	9.6	No	DHS (corrected from 9.3 to 9.6 after checking DHS report)	https://dhsprogram.com/		No
**BFA 2018**	15-49	11	No	WHO VAW 2018	https://www.who.int/publications/i/item/9789240022256		No
**BGD 2003**	15-49	31.05	No	WHO multi-country study (Garcia-Moreno et al., 2006)	https://www.who.int/gender/violence/who_multicountry_study/media_corner/Prevalence_intimatepartner_WHOStudy.pdf		Yes - based on averaged estimates for 2 or more locations
**BGD 2007**	15-49	23.9	No	DHS (via UN Women matrix)	https://www.endvawnow.org/uploads/browser/files/vawprevalence_matrix_june2013.pdf		Yes - based on averaged estimates for 2 or more locations
**BGD 2015**	15+	28.8	Yes	Calculated by UNFPA based on available country data from the Violence against Women Survey 2015	https://unstats.un.org/sdgs/indicators/database/		Yes - based on one estimate only
**BGD 2018**	15-49	23	No	WHO VAW 2018	https://www.who.int/publications/i/item/9789240022256		No
**BGR 2012**	18-74	9	Yes	calculated by FRA based on available country data from the 2012 FRA Violence against women: an EU-wide Survey	https://unstats.un.org/sdgs/indicators/database/		No
**BGR 2018**	15-49	6	No	WHO VAW 2018	https://www.who.int/publications/i/item/9789240022256		No
**BIH 2018**	18-74	3.4	No	Other (via UN Women Global Database on Violence against Women)	https://evaw-global-database.unwomen.org/en	Organization for Security and Co-operation in Europe (OSCE). 2019. OESCE-led Survey on Violence against Women: Main Report. Vienna, Austria: OSCE Secretariat.	Yes - based on averaged estimates for 2 or more locations
**BLR 2018**	15-49	6	No	WHO VAW 2018	https://www.who.int/publications/i/item/9789240022256		No
**BLZ 2018**	15-49	8	No	WHO VAW 2018	https://www.who.int/publications/i/item/9789240022256		No
**BOL 2008**	15-49	25.2	No	DHS data reported in PAHO 2012 publication VAW in LAC: Comparative analysis of population-based data from 12 countries	https://iris.paho.org/handle/10665.2/3471		No
**BOL 2016**	15+	27.1	Yes	PAHO 2018 publication Intimate Partner Violence against Women in the Americas: calculated by PAHO based on available country data from the Encuesta de Prevalencia y Caracteristicas de la Violencia contra las Mujeres 2016	https://unstats.un.org/sdgs/indicators/database/		Yes - based on averaged estimates for 2 or more locations
**BOL 2018**	15-49	18	No	WHO VAW 2018	https://www.who.int/publications/i/item/9789240022256		No
**BRA 2003**	15-49	12.0	No	WHO multi-country	https://www.who.int/gender/viole		Yes - based

		5		study (Garcia-Moreno et al., 2006)	nce/who_multicountry_study/media_corner/Prevalence_intimatepartner_WHOStudy.pdf		on averaged estimates for 2 or more locations
**BRA 2017**	16+	3.1	No	Other (via UN Women Global Database on Violence against Women)	https://evaw-global-database.unwomen.org/en	Bott S, Guedes A, Ruiz-Celis AP, Mendoza JA. Intimate partner violence in the Americas: A systematic review and reanalysis of national prevalence estimates. Based on data from: Instituto de Pesquisa DataSenado. 2018. Violência Doméstica e familiar contra a mulher: Pesquisa DataSenado. Brasilia: Instituto de Pesquisa DataSenado, Observatório da Mulher contra a Violência.	No
**BRA 2018**	15-49	6	No	WHO VAW 2018	https://www.who.int/publications/i/item/9789240022256		No
**BTN 2017**	15-64	6.1	No	Other (via UN Women Global Database on Violence against Women)	https://evaw-global-database.unwomen.org/en	National Commission for Women and Children. 2019. National Study on Women's Health and Life Experiences 2017.	Yes - based on averaged estimates for 2 or more locations
**BTN 2018**	15-49	9	No	WHO VAW 2018	https://www.who.int/publications/i/item/9789240022256		No
**BWA 2018**	15-49	17	No	WHO VAW 2018	https://www.who.int/publications/i/item/9789240022256		No
**CAF 2006**	15-49	26.3	Yes	MICS	https://unstats.un.org/sdgs/indicators/database/		No
**CAF 2018**	15-49	21	No	WHO VAW 2018	https://www.who.int/publications/i/item/9789240022256		No
**CAN 1993**	15+	3	No	Other		Statistics Canada. 2006. Measuring Violence Against Women Statistical Trends 2006. Statistics Canada. Ottawa.	No
**CAN 1999**	15+	3	No	Other		Statistics Canada, Canadian Centre for Justice Statistics. 2011. Family Violence in Canada: A Statistical Profile. Statistics Canada. Ottawa.	No
**CAN 2004**	15+	2	No	Other (via UN Women matrix)	https://www.endvawnow.org/uploads/browser/files/vawprevalence_matrix_june2013.pdf	Statistics Canada. 2006. Measuring Violence Against Women Statistical Trends 2006. Statistics Canada. Ottawa.	No
**CAN 2009**	15+	1.9	No	Other (via UN Women matrix)	https://www.endvawnow.org/uploads/browser/files/vawprevalence_matrix_june2013.pdf	Statistics Canada, Canadian Centre for Justice Statistics. 2011. Family Violence in Canada: A Statistical Profile. Statistics Canada. Ottawa.	No

**CAN 2018**	15-49	3	No	WHO VAW 2018	https://www.who.int/publications/i/item/9789240022256	No
**CHE 2003**	16+	1	No	IVAWS (via UN Women matrix, not confirmed)	https://www.endvawnow.org/uploads/browser/files/vawprevalence_matrix_june2013.pdf	No
**CHE 2018**	15-49	2	No	WHO VAW 2018	https://www.who.int/publications/i/item/9789240022256	No
**CHL 2018**	15-49	6	No	WHO VAW 2018	https://www.who.int/publications/i/item/9789240022256	No
**CHN 2013**	18-49	1.9	No	UNMCS	https://svri.org/sites/default/files/attachments/2018-05-09/Why%20do%20some%20men%20use%20violence%20against%20women_1.pdf	Yes - based on one estimate only
**CHN 2018**	15-49	8	No	WHO VAW 2018	https://www.who.int/publications/i/item/9789240022256	No
**CIV 2012**	15-49	22.9	No	DHS (corrected from 22 to 22.9 after checking report)	https://dhsprogram.com/	No
**CIV 2018**	15-49	16	No	WHO VAW 2018	https://www.who.int/publications/i/item/9789240022256	No
**CMR 2004**	15-49	28	No	DHS	https://dhsprogram.com/	No
**CMR 2011**	15-49	32.6	No	DHS	https://dhsprogram.com/	No
**CMR 2014**	15-49	32.7	Yes	MICS	https://unstats.un.org/sdgs/indicators/database/	No
**CMR 2018**	15-49	21.5	No	DHS	https://dhsprogram.com/	No
**COD 2007**	15-49	64.1	No	DHS	https://dhsprogram.com/	No
**COD 2014**	15-49	36.8	Yes	DHS	https://unstats.un.org/sdgs/indicators/database/	No
**COD 2018**	15-49	36	No	WHO VAW 2018	https://www.who.int/publications/i/item/9789240022256	No
**COK 2012**	15-64	9.4	Yes	The Cook Islands Family Health and Safety Study	https://unstats.un.org/sdgs/indicators/database/	No
**COK 2018**	15-49	14	No	WHO VAW 2018	https://www.who.int/publications/i/item/9789240022256	No
**COL 2005**	15-49	22.1	No	DHS data reported in PAHO 2012 publication VAW in LAC: Comparative analysis of population-based data from 12 countries	https://iris.paho.org/handle/10665.2/3471	No
**COL 2015**	15-49	18.4	Yes	DHS* (I couldn't find this estimate in the report)	https://unstats.un.org/sdgs/indicators/database/	No
**COL 2018**	15-49	12	No	WHO VAW 2018	https://www.who.int/publications/i/item/9789240022256	No
**COM 2012**	15-49	4.9	Yes	DHS	https://unstats.un.org/sdgs/indicators/database/	No
**COM 2018**	15-49	8	No	WHO VAW 2018	https://www.who.int/publications/i/item/9789240022256	No
**CPV 2005**	15-49	7.8	Yes	DHS	https://unstats.un.org/sdgs/indicators/database/	No
**CPV 2018**	15-49	11	No	WHO VAW 2018	https://www.who.int/publications/i/item/9789240022256	No
**CRI 2003**	16+	8	No	IVAWS	https://doi.org/10.1007/978-0-387-73204-6	No
**CRI 2018**	15-49	7	No	WHO VAW 2018	https://www.who.int/publications/i/item/9789240022256	No
**CUB 2018**	15-49	5	No	WHO VAW 2018	https://www.who.int/publications/i/item/9789240022256	No
**CYP 2012**	18-74	3	Yes	calculated by FRA based on available country data from the 2012 FRA Violence against women: an EU-wide Survey	https://unstats.un.org/sdgs/indicators/database/	No
**CYP 2018**	15-49	3	No	WHO VAW 2018	https://www.who.int/publications/i/item/9789240022256	No
**CZE 2003**	16+	9	No	IVAWS	https://doi.org/10.1007/978-0-387-73204-6	No
**CZE 2012**	18-74	6	Yes	calculated by FRA based on available country data from the	https://unstats.un.org/sdgs/indicators/database/	No

				2012 FRA Violence against women: an EU-wide Survey		
**CZE 2018**	15-49	4	No	WHO VAW 2018	https://www.who.int/publications/i/item/9789240022256	No
**DEU 2012**	18-74	5	Yes	calculated by FRA based on available country data from the 2012 FRA Violence against women: an EU-wide Survey	https://unstats.un.org/sdgs/indicators/database/	No
**DNK 2003**	16+	1	No	IVAWS	https://doi.org/10.1007/978-0-387-73204-6	No
**DNK 2012**	18-74	7	Yes	calculated by FRA based on available country data from the 2012 FRA Violence against women: an EU-wide Survey	https://unstats.un.org/sdgs/indicators/database/	No
**DNK 2018**	15-49	3	No	WHO VAW 2018	https://www.who.int/publications/i/item/9789240022256	No
**DOM 2002**	15-49	10.4	No	DHS	https://dhsprogram.com/	No
**DOM 2007**	15-49	11.7	No	DHS	https://dhsprogram.com/	No
**DOM 2013**	15-49	16	Yes	DHS	https://unstats.un.org/sdgs/indicators/database/	No
**DOM 2018**	15-49	10	No	WHO VAW 2018	https://www.who.int/publications/i/item/9789240022256	No
**ECU 2004**	15-49	11.1	No	RHS data reported in PAHO 2012 publication VAW in LAC: Comparative analysis of population-based data from 12 countries	https://iris.paho.org/handle/10665.2/3471	No
**ECU 2011**	15-49	10.8	Yes	PAHO 2018 publication Intimate Partner Violence against Women in the Americas: calculated by PAHO based on available country data from the 2011 Encuesta Nacional sobre Relaciones Familiares y Violencia de Género contra las Mujeres	https://unstats.un.org/sdgs/indicators/database/	No
**ECU 2018**	15-49	8	No	WHO VAW 2018	https://www.who.int/publications/i/item/9789240022256	No
**EGY 2005**	15-49	21.7	No	DHS	https://dhsprogram.com/	No
**EGY 2014**	15-49	14	Yes	DHS	https://unstats.un.org/sdgs/indicators/database/	No
**EGY 2018**	15-49	15	No	WHO VAW 2018	https://www.who.int/publications/i/item/9789240022256	No
**ESP 2012**	18-74	2	Yes	calculated by FRA based on available country data from the 2012 FRA Violence against women: an EU-wide Survey	https://unstats.un.org/sdgs/indicators/database/	No
**ESP 2018**	15-49	3	No	WHO VAW 2018	https://www.who.int/publications/i/item/9789240022256	No
**EST 2012**	18-74	4	Yes	calculated by FRA based on available country data from the 2012 FRA Violence against women: an EU-wide Survey	https://unstats.un.org/sdgs/indicators/database/	No
**EST 2018**	15-49	4	No	WHO VAW 2018	https://www.who.int/publications/i/item/9789240022256	No
**ETH 2003**	15-49	53.7	No	WHO multi-country study (Garcia-Moreno et al., 2006)	https://www.who.int/gender/violence/who_multicountry_study/media_corner/Prevalence_intimatepartner_WHOStudy.pdf	Yes - based on one estimate only
**ETH 2016**	15-49	19.8	Yes	DHS	https://unstats.un.org/sdgs/indicators/database/	No

**ETH 2018**	15-49	27	No	WHO VAW 2018	https://www.who.int/publications/i/item/9789240022256		No
**FIN 2012**	18-74	8	Yes	calculated by FRA based on available country data from the 2012 FRA Violence against women: an EU-wide Survey	https://unstats.un.org/sdgs/indicators/database/		No
**FIN 2018**	15-49	8	No	WHO VAW 2018	https://www.who.int/publications/i/item/9789240022256		No
**FJI 2011**	18-49	29.7	Yes	National Research on Women's Health and Life Experiences in Fiji (2010/2011)	https://unstats.un.org/sdgs/indicators/database/		No
**FJI 2018**	15-49	23	No	WHO VAW 2018	https://www.who.int/publications/i/item/9789240022256		No
**FRA 2000**	16+	10	No	IVAWS	https://doi.org/10.1007/978-0-387-73204-6		No
**FRA 2007**	18-59	3.3	No	Other (via UN Women Matrix; not clear from report if figure is actually for lifetime rather than last 12m)	https://www.endvawnow.org/uploads/browser/files/vawprevalence_matrix_june2013.pdf	Tournyol du Clos, L., Institut national des hautes études de sécurité, et Le Jeannic, T., division Conditions de vie des ménages, Insee. 2008. Les violences faites aux femmes. Insee Première N°1180. Paris.	No
**FRA 2012**	18-74	7	Yes	calculated by FRA based on available country data from the 2012 FRA Violence against women: an EU-wide Survey	https://unstats.un.org/sdgs/indicators/database/		No
**FRA 2018**	15-49	5	No	WHO VAW 2018	https://www.who.int/publications/i/item/9789240022256		No
**FSM 2014**	15-64	26	Yes	Federated States of Micronesia Family Health and Safety Study	https://unstats.un.org/sdgs/indicators/database/		No
**FSM 2018**	15-49	21	No	WHO VAW 2018	https://www.who.int/publications/i/item/9789240022256		No
**GAB 2012**	15-49	31.5	Yes	DHS	https://unstats.un.org/sdgs/indicators/database/		No
**GAB 2018**	15-49	22	No	WHO VAW 2018	https://www.who.int/publications/i/item/9789240022256		No
**GBR 2005**	16-59	5.9	No	Other* (via UN Women matrix but could not find equivalent statistic in report)	https://www.endvawnow.org/uploads/browser/files/vawprevalence_matrix_june2013.pdf	British Crime Survey	No
**GBR 2012**	18-74	6	Yes	calculated by FRA based on available country data from the 2012 FRA Violence against women: an EU-wide Survey	https://unstats.un.org/sdgs/indicators/database/		No
**GBR 2018**	15-49	4	No	WHO VAW 2018	https://www.who.int/publications/i/item/9789240022256		No
**GEO 2005**	15-44	2	No	RHS (via UN Women matrix)	https://www.endvawnow.org/uploads/browser/files/vawprevalence_matrix_june2013.pdf		No
**GEO 2018**	15-49	3	No	WHO VAW 2018	https://www.who.int/publications/i/item/9789240022256		No
**GHA 2008**	15-49	20	No	DHS (corrected from 19.2 to 20.0 after checking DHS report)	https://dhsprogram.com/		No
**GHA 2018**	15-49	10	No	WHO VAW 2018	https://www.who.int/publications/i/item/9789240022256		No
**GIN 2018**	15-49	21	No	WHO VAW 2018	https://www.who.int/publications/i/item/9789240022256		No
**GMB 2013**	15-49	7.3	Yes	DHS	https://unstats.un.org/sdgs/indicators/database/		No
**GMB 2018**	15-49	10	No	WHO VAW 2018	https://www.who.int/publications/i/item/9789240022256		No

**GNQ 2011**	15-49	43.6	Yes	DHS	https://unstats.un.org/sdgs/indicators/database/	No
**GNQ 2018**	15-49	29	No	WHO VAW 2018	https://www.who.int/publications/i/item/9789240022256	No
**GRC 2012**	18-74	8	Yes	calculated by FRA based on available country data from the 2012 FRA Violence against women: an EU-wide Survey	https://unstats.un.org/sdgs/indicators/database/	No
**GRC 2018**	15-49	5	No	WHO VAW 2018	https://www.who.int/publications/i/item/9789240022256	No
**GRD 2018**	15-49	8	No	WHO VAW 2018	https://www.who.int/publications/i/item/9789240022256	No
**GTM 2009**	15-49	9.9	No	RHS (via UN Women matrix)	https://www.endvawnow.org/uploads/browser/files/vawprevalence_matrix_june2013.pdf	No
**GTM 2015**	15-49	8.5	No	ENSMI (DHS) (Corrected from 8.9 to 8.5 after checking DHS report)	https://dhsprogram.com/	No
**GTM 2018**	15-49	7	No	WHO VAW 2018	https://www.who.int/publications/i/item/9789240022256	No
**GUY 2018**	15-49	10	No	WHO VAW 2018	https://www.who.int/publications/i/item/9789240022256	No
**HKG 2005**	16+	2	No	IVAWS	https://doi.org/10.1007/978-0-387-73204-6	No
**HKG 2018**	15-49	3	No	WHO VAW 2018	https://www.who.int/publications/i/item/9789240022256	No
**HND 2006**	15-49	9.9	No	DHS data reported in PAHO 2012 publication VAW in LAC: Comparative analysis of population-based data from 12 countries	https://iris.paho.org/handle/10665.2/3471	No
**HND 2012**	15-49	10.9	No	DHS (corrected from 11 to 10.9 after checking DHS report)	https://dhsprogram.com/	No
**HND 2018**	15-49	7	No	WHO VAW 2018	https://www.who.int/publications/i/item/9789240022256	No
**HRV 2012**	18-74	4	Yes	calculated by FRA based on available country data from the 2012 FRA Violence against women: an EU-wide Survey	https://unstats.un.org/sdgs/indicators/database/	No
**HRV 2018**	15-49	4	No	WHO VAW 2018	https://www.who.int/publications/i/item/9789240022256	No
**HTI 2000**	15-49	20.7	No	DHS	https://dhsprogram.com/	No
**HTI 2006**	15-49	16.8	No	DHS	https://dhsprogram.com/	No
**HTI 2012**	15-49	14.9	No	DHS	https://dhsprogram.com/	No
**HTI 2017**	15-49	13.9	Yes	DHS	https://unstats.un.org/sdgs/indicators/database/	No
**HTI 2018**	15-49	12	No	WHO VAW 2018	https://www.who.int/publications/i/item/9789240022256	No
**HUN 2012**	18-74	8	Yes	calculated by FRA based on available country data from the 2012 FRA Violence against women: an EU-wide Survey	https://unstats.un.org/sdgs/indicators/database/	No
**HUN 2018**	15-49	6	No	WHO VAW 2018	https://www.who.int/publications/i/item/9789240022256	No
**IDN 2018**	15-49	9	No	WHO VAW 2018	https://www.who.int/publications/i/item/9789240022256	No
**IND 2006**	15-49	23.9	No	DHS	https://dhsprogram.com/	No
**IND 2016**	15-49	23.7	No	NFHS (DHS) - corrected to 23.7 from 22 after checking report	https://dhsprogram.com/	No
**IND 2018**	15-49	18	No	WHO VAW 2018	https://www.who.int/publications/i/item/9789240022256	No
**IRL 2012**	18-74	4	Yes	calculated by FRA based on available country data from the	https://unstats.un.org/sdgs/indicators/database/	No

				2012 FRA Violence against women: an EU-wide Survey			
**IRL 2018**	15-49	3	No	WHO VAW 2018	https://www.who.int/publications/i/item/9789240022256		No
**IRN 2018**	15-49	18	No	WHO VAW 2018	https://www.who.int/publications/i/item/9789240022256		No
**ISL 2008**	18-80	2	No	Other (via UN Women Global Database on Violence against Women)	https://evaw-global-database.unwomen.org/en	Ingólfur Gíslason, 2008. Ofbeldi í nánum samböndum: orsakir, afleiðingar, úrræði [Violence in intimate relationships: causes, consequences, remedies]. Reykjavík: Félagsmálaráðuneytið (Ministry of Social Affairs – now Ministry for Welfare)	No
**ISL 2018**	15-49	3	No	WHO VAW 2018	https://www.who.int/publications/i/item/9789240022256		No
**ISR 2018**	15-49	6	No	WHO VAW 2018	https://www.who.int/publications/i/item/9789240022256		No
**ITA 2006**	16+	2.4	No	IVAWS	https://doi.org/10.1007/978-0-387-73204-6		No
**ITA 2012**	18-74	7	Yes	calculated by FRA based on available country data from the 2012 FRA Violence against women: an EU-wide Survey	https://unstats.un.org/sdgs/indicators/database/		No
**ITA 2018**	15-49	4	No	WHO VAW 2018	https://www.who.int/publications/i/item/9789240022256		No
**JAM 2009**	15-49	7.7	No	RHS (via UN Women matrix)	https://www.endvawnow.org/uploads/browser/files/vawprevalence_matrix_june2013.pdf		No
**JAM 2016**	15-64	8.52	Yes	Calculated by UN Women based on available country data from the Women's Health Survey 2016	https://unstats.un.org/sdgs/indicators/database/		No
**JAM 2018**	15-49	7	No	WHO VAW 2018	https://www.who.int/publications/i/item/9789240022256		No
**JOR 2007**	15-49	14.6	No	DHS	https://dhsprogram.com/		No
**JOR 2012**	15-49	14.1	Yes	DHS	https://unstats.un.org/sdgs/indicators/database/		No
**JOR 2018**	15-49	13.8	No	DHS	https://dhsprogram.com/		No
**JPN 2003**	18-49	3.8	No	WHO multi-country study (Garcia-Moreno et al., 2006)	https://www.who.int/gender/violence/who_multicountry_study/media_corner/Prevalence_intimatepartner_WHOStudy.pdf		Yes - based on one estimate only
**JPN 2018**	15-49	4	No	WHO VAW 2018	https://www.who.int/publications/i/item/9789240022256		No
**KAZ 2011**	15-49	15.7	No	MICS	http://mics.unicef.org/surveys		No
**KAZ 2015**	18-75	4.7	No	Other (via UN Women Global Database on Violence against Women)	https://evaw-global-database.unwomen.org/en	Statistics Committee of the Ministry of National Economy. 2017. Sample Survey on Violence Against Women in Kazakhstan. Astana, Kazakhstan: Statistics Committee of the Ministry of National Economy of RK.	No
**KAZ 2018**	15-49	6	No	WHO VAW 2018	https://www.who.int/publications/i/item/9789240022256		No
**KEN 2003**	15-49	28.2	No	DHS	https://dhsprogram.com/		No
**KEN 2009**	15-49	34	No	DHS	https://dhsprogram.com/		No
**KEN 2014**	15-49	25.5	Yes	DHS	https://unstats.un.org/sdgs/indicat		No

					ors/database/		
**KEN 2018**	15-49	23	No	WHO VAW 2018	https://www.who.int/publications/i/item/9789240022256		No
**KGZ 2012**	15-49	17.1	Yes	DHS	https://unstats.un.org/sdgs/indicators/database/		No
**KGZ 2018**	15-49	13	No	WHO VAW 2018	https://www.who.int/publications/i/item/9789240022256		No
**KHM 2000**	15-49	15.2	No	DHS	https://dhsprogram.com/		No
**KHM 2005**	15-49	8.7	No	DHS	https://dhsprogram.com/		No
**KHM 2013**	18-49	3.2	No	UNMCS	https://svri.org/sites/default/files/attachments/2018-05-09/Why%20do%20some%20men%20use%20violence%20against%20women_1.pdf		No
**KHM 2014**	15-49	10.9	Yes	DHS	https://unstats.un.org/sdgs/indicators/database/		No
**KHM 2018**	15-49	9	No	WHO VAW 2018	https://www.who.int/publications/i/item/9789240022256		No
**KIR 2008**	15-49	36.1	Yes	Family Health and Support Study	https://unstats.un.org/sdgs/indicators/database/		No
**KIR 2018**	15-49	25	No	WHO VAW 2018	https://www.who.int/publications/i/item/9789240022256		No
**KIR 2019**	15-49	43.4	No	MICS	http://mics.unicef.org/surveys		No
**KOR 2018**	15-49	8	No	WHO VAW 2018	https://www.who.int/publications/i/item/9789240022256		No
**LAO 2014**	15-64	6.4	Yes	Lao National Survey on Women's Health and Life Experiences	https://unstats.un.org/sdgs/indicators/database/		No
**LAO 2018**	15-49	8	No	WHO VAW 2018	https://www.who.int/publications/i/item/9789240022256		No
**LBR 2007**	15-49	36.3	No	DHS (corrected from 35to 36.3 after checking DHS report)	https://dhsprogram.com/		No
**LBR 2018**	15-49	27	No	WHO VAW 2018	https://www.who.int/publications/i/item/9789240022256		No
**LKA 2013**	18-49	1.5	No	UNMCS	https://svri.org/sites/default/files/attachments/2018-05-09/Why%20do%20some%20men%20use%20violence%20against%20women_1.pdf		No
**LKA 2018**	15-49	4	No	WHO VAW 2018	https://www.who.int/publications/i/item/9789240022256		No
**LSO 2018**	15-49	16	No	WHO VAW 2018	https://www.who.int/publications/i/item/9789240022256		No
**LTU 2012**	18-74	6	Yes	calculated by FRA based on available country data from the 2012 FRA Violence against women: an EU-wide Survey	https://unstats.un.org/sdgs/indicators/database/		No
**LTU 2018**	15-49	5	No	WHO VAW 2018	https://www.who.int/publications/i/item/9789240022256		No
**LUX 2012**	18-74	3	Yes	calculated by FRA based on available country data from the 2012 FRA Violence against women: an EU-wide Survey	https://unstats.un.org/sdgs/indicators/database/		No
**LUX 2018**	15-49	4	No	WHO VAW 2018	https://www.who.int/publications/i/item/9789240022256		No
**LVA 2012**	18-74	7	Yes	calculated by FRA based on available country data from the 2012 FRA Violence against women: an EU-wide Survey	https://unstats.un.org/sdgs/indicators/database/		No
**LVA 2018**	15-49	6	No	WHO VAW 2018	https://www.who.int/publications/i/item/9789240022256		No
**MAR 2010**	18-64	11.5	No	Other (via UN Women matrix)	https://www.endvawnow.org/uploads/browser/files/vawprevalence_matrix_june2013.pdf	Morrocan High Commission of Planning. 2012. Enquête nationale sur la prévalence de la violence à l'égard des femmes au	No

						Maroc: 2009. Morrocan High Commission of Planning. Rabat.	
**MAR 2018**	15-49	10	No	WHO VAW 2018	https://www.who.int/publications/i/item/9789240022256		No
**MDA 2005**	15-49	14.6	Yes	DHS	https://unstats.un.org/sdgs/indicators/database/		No
**MDA 2018**	15-49	9	No	WHO VAW 2018	https://www.who.int/publications/i/item/9789240022256		No
**MDG 2018**	15-49	16	No	MICS	http://mics.unicef.org/surveys		No
**MDV 2006**	15-49	6.4	Yes	Study on Women's Health and Life Experiences	https://unstats.un.org/sdgs/indicators/database/		No
**MDV 2016**	15-49	5.6	No	DHS	https://dhsprogram.com/		No
**MDV 2018**	15-49	6	No	WHO VAW 2018	https://www.who.int/publications/i/item/9789240022256		No
**MEX 2016**	15+	9.5	Yes	PAHO 2018 publication Intimate Partner Violence against Women in the Americas: calculated by PAHO based on available country data from the 2016 ENDIREH	https://unstats.un.org/sdgs/indicators/database/		No
**MEX 2018**	15-49	10	No	WHO VAW 2018	https://www.who.int/publications/i/item/9789240022256		No
**MHL 2007**	15-49	24.7	No	DHS (via UN Women matrix)	https://www.endvawnow.org/uploads/browser/files/vawprevalence_matrix_june2013.pdf		No
**MHL 2014**	15-64	20.1	Yes	Republic of the Marshall Islands National Study on Family Health and Safety	https://unstats.un.org/sdgs/indicators/database/		No
**MHL 2018**	15-49	19	No	WHO VAW 2018	https://www.who.int/publications/i/item/9789240022256		No
**MKD 2018**	15-49	4	No	WHO VAW 2018	https://www.who.int/publications/i/item/9789240022256		No
**MLI 2006**	15-49	21.5	Yes	DHS* (I couldn't find this in DHS report)	https://unstats.un.org/sdgs/indicators/database/		No
**MLI 2013**	15-49	26.9	No	DHS	https://dhsprogram.com/		No
**MLI 2018**	15-49	20.9	No	DHS	https://dhsprogram.com/		No
**MLT 2010**	18-59	13.6	No	WHO (UN Women matrix) / Study on the Prevalence of Domestic Violence against Women in Malta*	https://www.endvawnow.org/uploads/browser/files/vawprevalence_matrix_june2013.pdf		No
**MLT 2012**	18-74	5	Yes	calculated by FRA based on available country data from the 2012 FRA Violence against women: an EU-wide Survey	https://unstats.un.org/sdgs/indicators/database/		No
**MLT 2018**	15-49	4	No	WHO VAW 2018	https://www.who.int/publications/i/item/9789240022256		No
**MMR 2016**	15-49	11	Yes	DHS	https://unstats.un.org/sdgs/indicators/database/		No
**MMR 2018**	15-49	11	No	WHO VAW 2018	https://www.who.int/publications/i/item/9789240022256		No
**MNE 2018**	15-49	4	No	WHO VAW 2018	https://www.who.int/publications/i/item/9789240022256		No
**MNG 2017**	15-64	14.7	Yes	2017 National Study on Gender-based violence in Mongolia	https://unstats.un.org/sdgs/indicators/database/		No
**MNG 2018**	15-49	12	No	WHO VAW 2018	https://www.who.int/publications/i/item/9789240022256		No
**MOZ 2004**	16+	18	No	IVAWS	https://doi.org/10.1007/978-0-387-73204-6		No
**MOZ 2011**	15-49	27.7	No	DHS	https://dhsprogram.com/		No

**MOZ 2015**	18-49	15.5	Yes	AIS: Inquérito de Indicadores de Imunização, Malária e HIV/SIDA	https://unstats.un.org/sdgs/indicators/database/		No
**MOZ 2018**	15-49	16	No	WHO VAW 2018	https://www.who.int/publications/i/item/9789240022256		No
**MWI 2004**	15-49	18.5	No	DHS	https://dhsprogram.com/		No
**MWI 2010**	15-49	22.1	No	DHS	https://dhsprogram.com/		No
**MWI 2016**	15-49	24.3	Yes	DHS	https://unstats.un.org/sdgs/indicators/database/		No
**MWI 2018**	15-49	17	No	WHO VAW 2018	https://www.who.int/publications/i/item/9789240022256		No
**NAM 2003**	15-49	19.5	No	WHO multi-country study (Garcia-Moreno et al., 2006)	https://www.who.int/gender/violence/who_multicountry_study/media_corner/Prevalence_intimatepartner_WHOStudy.pdf		Yes - based on one estimate only
**NAM 2013**	15-49	20.2	Yes	DHS	https://unstats.un.org/sdgs/indicators/database/		No
**NAM 2018**	15-49	16	No	WHO VAW 2018	https://www.who.int/publications/i/item/9789240022256		No
**NER 2018**	15-49	13	No	WHO VAW 2018	https://www.who.int/publications/i/item/9789240022256		No
**NGA 2008**	15-49	15.2	No	DHS	https://dhsprogram.com/		No
**NGA 2013**	15-49	11	Yes	DHS	https://unstats.un.org/sdgs/indicators/database/		No
**NGA 2018**	15-49	13.8	No	DHS	https://dhsprogram.com/		No
**NIC 2007**	15-49	9.3	No	RHS (via UN Women matrix)	https://www.endvawnow.org/uploads/browser/files/vawprevalence_matrix_june2013.pdf		No
**NIC 2012**	15-49	7.5	Yes	ENDESA	https://unstats.un.org/sdgs/indicators/database/		No
**NIC 2018**	15-49	6	No	WHO VAW 2018	https://www.who.int/publications/i/item/9789240022256		No
**NLD 2012**	18-74	7	Yes	calculated by FRA based on available country data from the 2012 FRA Violence against women: an EU-wide Survey	https://unstats.un.org/sdgs/indicators/database/		No
**NLD 2018**	15-49	5	No	WHO VAW 2018	https://www.who.int/publications/i/item/9789240022256		No
**NOR 2008**	20-55	5.5	No	Other (via UN Women matrix)	https://www.endvawnow.org/uploads/browser/files/vawprevalence_matrix_june2013.pdf	Neroien A, Schei B. 2008. Partner violence and health: results from the first national study on violence against women in Norway. Scandanavian Journal of Public Health 36(2): 161-8.	No
**NOR 2018**	15-49	4	No	WHO VAW 2018	https://www.who.int/publications/i/item/9789240022256		No
**NPL 2011**	15-49	14.3	No	DHS	https://dhsprogram.com/		No
**NPL 2016**	15-49	11.2	Yes	DHS	https://unstats.un.org/sdgs/indicators/database/		No
**NPL 2018**	15-49	11	No	WHO VAW 2018	https://www.who.int/publications/i/item/9789240022256		No
**NRU 2013**	15-64	22	No	Other (via UN Women Global Database on Violence against Women)	https://evaw-global-database.unwomen.org/en	Carlued Leon and Tara Detogia, 2014. Nauru Family Health and Support Study - An Exploratory Study on Violence against Women.	No
**NRU 2018**	15-49	20	No	WHO VAW 2018	https://www.who.int/publications/i/item/9789240022256		No
**NZL 2002**	18-64	5.55	No	WHO multi-country study (UN matrix)	https://www.who.int/gender/violence/who_multicountry_study/media_corner/Prevalence_intimatepartner_WHOStudy.pdf		Yes - based on averaged estimates for 2 or more locations
**NZL 2018**	15-49	4	No	WHO VAW 2018	https://www.who.int/publications/		No

					i/item/9789240022256	
**PAK 2018**	15-49	14.5	No	DHS	https://dhsprogram.com/	No
**PAN 2009**	15-49	10.1	Yes	PAHO 2018 publication Intimate Partner Violence against Women in the Americas: calculated by PAHO based on available country data from the 2009 Encuesta Nacional de Salud Sexual y Reproductiva Panama 2009	https://unstats.un.org/sdgs/indicators/database/	No
**PAN 2018**	15-49	8	No	WHO VAW 2018	https://www.who.int/publications/i/item/9789240022256	No
**PER 2003**	15-49	26.7	No	WHO multi-country study (Garcia-Moreno et al., 2006)	https://www.who.int/gender/violence/who_multicountry_study/media_corner/Prevalence_intimatepartner_WHOStudy.pdf	Yes - based on averaged estimates for 2 or more locations
**PER 2008**	15-49	14.9	No	DHS	https://dhsprogram.com/	No
**PER 2009**	15-49	14.2	No	DHS	https://dhsprogram.com/	No
**PER 2010**	15-49	13.9	No	DHS	https://dhsprogram.com/	No
**PER 2011**	15-49	13.6	No	DHS	https://dhsprogram.com/	No
**PER 2012**	15-49	12.9	No	DHS	https://dhsprogram.com/	No
**PER 2013**	15-49	12.1	No	DHS	https://dhsprogram.com/	No
**PER 2014**	15-49	12.8	No	DHS	https://dhsprogram.com/	No
**PER 2015**	15-49	11.7	No	Encuesta demografica y de salud familiar ENDES	https://proyectos.inei.gob.pe/endes/	
**PER 2016**	15-49	10.8	Yes	Encuesta demografica y de salud familiar ENDES	https://unstats.un.org/sdgs/indicators/database/	No
**PER 2017**	15-49	10.6	No	Encuesta demografica y de salud familiar ENDES	https://proyectos.inei.gob.pe/endes/	
**PER 2018**	15-49	10.9	No	Encuesta demografica y de salud familiar ENDES	https://proyectos.inei.gob.pe/endes/	
**PER 2019**	15-49	10	No	Encuesta demografica y de salud familiar ENDES	https://proyectos.inei.gob.pe/endes/	
**PHL 2005**	16+	3	No	IVAWS	https://doi.org/10.1007/978-0-387-73204-6	No
**PHL 2008**	15-49	10.3	No	DHS	https://dhsprogram.com/	No
**PHL 2013**	15-49	7.1	No	DHS	https://dhsprogram.com/	No
**PHL 2017**	15-49	5.5	Yes	DHS	https://unstats.un.org/sdgs/indicators/database/	No
**PHL 2018**	15-49	6	No	WHO VAW 2018	https://www.who.int/publications/i/item/9789240022256	No
**PLW 2013**	15-64	9.6	Yes	Belau Family Health and Safety Study	https://unstats.un.org/sdgs/indicators/database/	No
**PLW 2018**	15-49	14	No	WHO VAW 2018	https://www.who.int/publications/i/item/9789240022256	No
**PNG 2013**	18-49	13.4	No	UNMCS	https://svri.org/sites/default/files/attachments/2018-05-09/Why%20do%20some%20men%20use%20violence%20against%20women_1.pdf	Yes - based on one estimate only
**PNG 2018**	15-49	47.6	No	DHS	https://dhsprogram.com/	No
**POL 2004**	16+	3	No	IVAWS	https://doi.org/10.1007/978-0-387-73204-6	No
**POL 2012**	18-74	3	Yes	calculated by FRA based on available country data from the 2012 FRA Violence against women: an EU-wide Survey	https://unstats.un.org/sdgs/indicators/database/	No
**POL 2018**	15-49	3	No	WHO VAW 2018	https://www.who.int/publications/i/item/9789240022256	No
**PRT 2012**	18-74	6	Yes	calculated by FRA based on available country data from the 2012 FRA Violence against women: an EU-	https://unstats.un.org/sdgs/indicators/database/	No

				wide Survey			
**PRT 2018**	15-49	4	No	WHO VAW 2018	https://www.who.int/publications/i/item/9789240022256		No
**PRY 2008**	15-44	8	No	RHS data reported in PAHO 2012 publication VAW in LAC: Comparative analysis of population-based data from 12 countries	https://iris.paho.org/handle/10665.2/3471		No
**PRY 2018**	15-49	6	No	WHO VAW 2018	https://www.who.int/publications/i/item/9789240022256		No
**PSE 2018**	15-49	19	No	WHO VAW 2018	https://www.who.int/publications/i/item/9789240022256		No
**ROU 2012**	18-74	7	Yes	calculated by FRA based on available country data from the 2012 FRA Violence against women: an EU-wide Survey	https://unstats.un.org/sdgs/indicators/database/		No
**ROU 2018**	15-49	7	No	WHO VAW 2018	https://www.who.int/publications/i/item/9789240022256		No
**RWA 2005**	15-49	25.6	No	DHS	https://dhsprogram.com/		No
**RWA 2010**	15-49	44.3	No	DHS	https://dhsprogram.com/		No
**RWA 2015**	15-49	20.7	Yes	DHS	https://unstats.un.org/sdgs/indicators/database/		No
**RWA 2018**	15-49	23	No	WHO VAW 2018	https://www.who.int/publications/i/item/9789240022256		No
**SDN 2018**	15-49	17	No	WHO VAW 2018	https://www.who.int/publications/i/item/9789240022256		No
**SEN 2017**	15-49	12.2	Yes	DHS	https://unstats.un.org/sdgs/indicators/database/		No
**SEN 2018**	15-49	12	No	WHO VAW 2018	https://www.who.int/publications/i/item/9789240022256		No
**SGP 2009**	18-69	1	No	Other (via UN Women Global Database on Violence against Women)	https://evaw-global-database.unwomen.org/en	Bouhours, B., Cheong, C.W., Bong, B. and Anderson, S., 2013. International Violence Against Women Survey: Final Report on Singapore.	No
**SGP 2018**	15-49	2	No	WHO VAW 2018	https://www.who.int/publications/i/item/9789240022256		No
**SLB 2008**	15-49	41.8	Yes	Solomon Islands Family Health and Safety Study: A study on violence against women and children	https://unstats.un.org/sdgs/indicators/database/		No
**SLB 2018**	15-49	28	No	WHO VAW 2018	https://www.who.int/publications/i/item/9789240022256		No
**SLE 2013**	15-49	28.7	Yes	DHS	https://unstats.un.org/sdgs/indicators/database/		No
**SLE 2018**	15-49	20	No	WHO VAW 2018	https://www.who.int/publications/i/item/9789240022256		No
**SLV 2008**	15-49	7.7	No	RHS (via UN Women matrix)	https://www.endvawnow.org/uploads/browser/files/vawprevalence_matrix_june2013.pdf		No
**SLV 2014**	15-64	6.7	Yes	PAHO 2018 publication Intimate Partner Violence against Women in the Americas: calculated by PAHO based on available country data from the Violencia Contra las Mujeres en El Salvador: Estudio poblacional 2014	https://unstats.un.org/sdgs/indicators/database/		No
**SLV 2018**	15-49	6	No	WHO VAW 2018	https://www.who.int/publications/i/item/9789240022256		No
**SRB 2003**	15-49	3.7	No	WHO multi-country study (Garcia-Moreno et al., 2006)	https://www.who.int/gender/violence/who_multicountry_study/media_corner/Prevalence_intimatepa		Yes - based on one estimate only

					rtner_WHOStudy.pdf		
**SRB 2018**	18-74	3.4	No	Other (via UN Women Global Database on Violence against Women)	https://evaw-global-database.unwomen.org/en	Organization for Security and Co-operation in Europe (OSCE). 2019. OESCE-led Survey on Violence against Women: Main Report. Vienna, Austria: OSCE Secretariat.	No
**SSD 2018**	15-49	27	No	WHO VAW 2018	https://www.who.int/publications/i/item/9789240022256		No
**STP 2009**	15-49	27.9	No	DHS (changed UN SDG value of 26.3 to 27.9 to match DHS report and UN matrix)	https://dhsprogram.com/		No
**STP 2018**	15-49	18	No	WHO VAW 2018	https://www.who.int/publications/i/item/9789240022256		No
**SUR 2018**	15-49	8	No	WHO VAW 2018	https://www.who.int/publications/i/item/9789240022256		No
**SVK 2008**	?	12.2	No	Other* (via UN Women matrix; can't verify as can't find report))	https://www.endvawnow.org/uploads/browser/files/vawprevalence_matrix_june2013.pdf	Bondarova et al., 2008 "Representative Research on Prevalence and experience of Women with Violence against Women in Slovakia". Institute for Labor and Family Research, Bratislava	No
**SVK 2012**	18-74	8	Yes	calculated by FRA based on available country data from the 2012 FRA Violence against women: an EU-wide Survey	https://unstats.un.org/sdgs/indicators/database/		No
**SVK 2018**	15-49	6	No	WHO VAW 2018	https://www.who.int/publications/i/item/9789240022256		No
**SVN 2012**	18-74	2	Yes	calculated by FRA based on available country data from the 2012 FRA Violence against women: an EU-wide Survey	https://unstats.un.org/sdgs/indicators/database/		No
**SVN 2018**	15-49	3	No	WHO VAW 2018	https://www.who.int/publications/i/item/9789240022256		No
**SWE 2012**	18-74	6	Yes	calculated by FRA based on available country data from the 2012 FRA Violence against women: an EU-wide Survey	https://unstats.un.org/sdgs/indicators/database/		No
**SWE 2018**	15-49	6	No	WHO VAW 2018	https://www.who.int/publications/i/item/9789240022256		No
**SWZ 2018**	15-49	18	No	WHO VAW 2018	https://www.who.int/publications/i/item/9789240022256		No
**TCD 2015**	15-49	17.5	Yes	DHS	https://unstats.un.org/sdgs/indicators/database/		No
**TCD 2018**	15-49	16	No	WHO VAW 2018	https://www.who.int/publications/i/item/9789240022256		No
**TGO 2014**	15-49	12.7	Yes	DHS	https://unstats.un.org/sdgs/indicators/database/		No
**TGO 2018**	15-49	13	No	WHO VAW 2018	https://www.who.int/publications/i/item/9789240022256		No
**THA 2003**	15-49	22.1	No	WHO multi-country study (Garcia-Moreno et al., 2006)	https://www.who.int/gender/violence/who_multicountry_study/media_corner/Prevalence_intimatepartner_WHOStudy.pdf		Yes - based on averaged estimates for 2 or more locations
**THA 2018**	15-49	9	No	WHO VAW 2018	https://www.who.int/publications/i/item/9789240022256		No

**TJK 2012**	15-49	15.2	No	DHS	https://dhsprogram.com/	No
**TJK 2017**	15-49	19	Yes	DHS	https://unstats.un.org/sdgs/indicators/database/	No
**TJK 2018**	15-49	14	No	WHO VAW 2018	https://www.who.int/publications/i/item/9789240022256	No
**TLS 2010**	15-49	31.5	No	DHS	https://dhsprogram.com/	No
**TLS 2016**	15-49	34.6	Yes	DHS	https://unstats.un.org/sdgs/indicators/database/	No
**TLS 2018**	15-49	28	No	WHO VAW 2018	https://www.who.int/publications/i/item/9789240022256	No
**TON 2009**	15-49	18.9	Yes	National Study on Domestic Violence against Women in Tonga	https://unstats.un.org/sdgs/indicators/database/	No
**TON 2018**	15-49	17	No	WHO VAW 2018	https://www.who.int/publications/i/item/9789240022256	No
**TTO 2017**	15-64	5.7	Yes	National Women's and Health Survey for Trinidad and Tobago. NB: corrected this from UN Women calculation provided in SDG figures to match that in report	https://unstats.un.org/sdgs/indicators/database/	No
**TTO 2018**	15-49	8	No	WHO VAW 2018	https://www.who.int/publications/i/item/9789240022256	No
**TUN 2018**	15-49	10	No	WHO VAW 2018	https://www.who.int/publications/i/item/9789240022256	No
**TUR 2008**	15-59	13.7	No	WHO (UN Women matrix) / National Research on Domestic Violence against Women in Turkey	https://www.endvawnow.org/uploads/browser/files/vawprevalence_matrix_june2013.pdf	No
**TUR 2014**	15-59	11	Yes	Domestic Violence against Women in Turkey	https://unstats.un.org/sdgs/indicators/database/	No
**TUR 2018**	15-49	12	No	WHO VAW 2018	https://www.who.int/publications/i/item/9789240022256	No
**TUV 2007**	15-49	25	Yes	DHS	https://unstats.un.org/sdgs/indicators/database/	No
**TUV 2018**	15-49	20	No	WHO VAW 2018	https://www.who.int/publications/i/item/9789240022256	No
**TZA 2003**	15-49	25.3	No	WHO multi-country study (Garcia-Moreno et al., 2006)	https://www.who.int/gender/violence/who_multicountry_study/media_corner/Prevalence_intimatepartner_WHOStudy.pdf	Yes - based on averaged estimates for 2 or more locations
**TZA 2010**	15-49	36.8	No	DHS	https://dhsprogram.com/	No
**TZA 2016**	15-49	29.6	Yes	DHS	https://unstats.un.org/sdgs/indicators/database/	No
**TZA 2018**	15-49	24	No	WHO VAW 2018	https://www.who.int/publications/i/item/9789240022256	No
**UGA 2006**	15-49	45	No	DHS	https://dhsprogram.com/	No
**UGA 2011**	15-49	34.6	No	DHS	https://dhsprogram.com/	No
**UGA 2016**	15-49	29.9	Yes	DHS	https://unstats.un.org/sdgs/indicators/database/	No
**UGA 2018**	15-49	26	No	WHO VAW 2018	https://www.who.int/publications/i/item/9789240022256	No
**UKR 2007**	15-49	10.6	No	DHS (corrected from 10.2 to 10.6 after checking DHS report)	https://dhsprogram.com/	No
**UKR 2018**	15-49	9	No	WHO VAW 2018	https://www.who.int/publications/i/item/9789240022256	No
**URY 2013**	15+	2.8	Yes	PAHO 2018 publication Intimate Partner Violence against Women in the Americas: calculated by PAHO based on available country data from the 2013 Encuesta Nacional de Violencia basada en Genero y Generaciones	https://unstats.un.org/sdgs/indicators/database/	No
**URY 2018**	15-49	4	No	WHO VAW 2018	https://www.who.int/publications/	No

					i/item/9789240022256	
**USA 2000**	18+	1.5	No	RHS (via UN Women matrix)	https://www.endvawnow.org/uploads/browser/files/vawprevalence_matrix_june2013.pdf	No
**USA 2018**	15-49	6	No	WHO VAW 2018	https://www.who.int/publications/i/item/9789240022256	No
**VEN 2018**	15-49	8	No	WHO VAW 2018	https://www.who.int/publications/i/item/9789240022256	No
**VNM 2010**	18-60	10.8	Yes	NATIONAL STUDY ON DOMESTIC VIOLENCE AGAINST WOMEN IN VIET NAM	https://unstats.un.org/sdgs/indicators/database/	No
**VNM 2018**	15-49	10	No	WHO VAW 2018	https://www.who.int/publications/i/item/9789240022256	No
**VUT 2009**	15-49	44	Yes	National Survey on Women's Lives and Family Relationships	https://unstats.un.org/sdgs/indicators/database/	No
**VUT 2018**	15-49	29	No	WHO VAW 2018	https://www.who.int/publications/i/item/9789240022256	No
**WSM 2003**	15-49	22.4	No	WHO multi-country study (Garcia-Moreno et al., 2006)	https://www.who.int/gender/violence/who_multicountry_study/media_corner/Prevalence_intimatepartner_WHOStudy.pdf	No
**WSM 2018**	15-49	18	No	WHO VAW 2018	https://www.who.int/publications/i/item/9789240022256	No
**WSM 2020**	15-49	22	Yes	The Samoa Family Health and Safety Study	https://unstats.un.org/sdgs/indicators/database/	No
**XKX 2018**	15-49	5	No	WHO VAW 2018	https://www.who.int/publications/i/item/9789240022256	No
**ZAF 2016**	15-49	8.5	No	DHS	https://dhsprogram.com/	No
**ZAF 2018**	15-49	13	No	WHO VAW 2018	https://www.who.int/publications/i/item/9789240022256	No
**ZMB 2007**	15-49	43	No	DHS	https://dhsprogram.com/	No
**ZMB 2014**	15-49	26.7	Yes	DHS	https://unstats.un.org/sdgs/indicators/database/	No
**ZMB 2018**	15-49	25.3	No	DHS	https://dhsprogram.com/	No
**ZWE 2006**	15-49	30.5	No	DHS	https://dhsprogram.com/	No
**ZWE 2011**	15-49	27.2	No	DHS	https://dhsprogram.com/	No
**ZWE 2015**	15-49	19.9	Yes	DHS	https://unstats.un.org/sdgs/indicators/database/	No
**ZWE 2018**	15-49	18	No	WHO VAW 2018	https://www.who.int/publications/i/item/9789240022256	No
**ZWE 2019**	15-49	18.7	No	MICS	http://mics.unicef.org/surveys	No

Appendix C. Table A3: Total sample in the main macro analysis and sensitivity analysis (analytical sample shown with sensitivity analysis sample in brackets)

Country	LCA analyses N = 5,732 (N = 1,451)		IPV analyses N = 3,175 (N = 134)	
	Number of country-years	Year range	Number of country-years	Year range
**Afghanistan**	31 (4)	1990-2020 (1995-2015)	16 (1)	2005-2020 (2015-2015)
**Angola**	31 (9)	1990-2020 (1990-2016)	15 (1)	2006-2020 (2016-2016)
**Albania**	31 (7)	1990-2020 (1995-2018)	13 (1)	2008-2020 (2018-2018)
**United Arab Emirates**	31 (7)	1990-2020 (1990-2017)	0 (0)	- (-)
**Argentina**	31 (11)	1990-2020 (1990-2018)	16 (2)	2005-2020 (2015-2018)
**Armenia**	31 (8)	1990-2020 (1990-2016)	15 (1)	2006-2020 (2016-2016)
**Antigua and Barbuda**	31 (6)	1990-2020 (1990-2015)	0 (0)	- (-)
**Australia**	31 (8)	1990-2020 (1990-2018)	28 (1)	1993-2020 (2018-2018)
**Austria**	31 (6)	1990-2020 (1990-2015)	19 (0)	2002-2020 (-)
**Azerbaijan**	31 (7)	1990-2020 (1990-2015)	25 (0)	1996-2020 (-)
**Burundi**	31 (12)	1990-2020 (1990-2017)	14 (1)	2007-2020 (2017-2017)
**Belgium**	31 (6)	1990-2020 (1990-2015)	19 (0)	2002-2020 (-)
**Benin**	31 (11)	1990-2020 (1990-2018)	13 (1)	2008-2020 (2018-2018)
**Burkina Faso**	31 (13)	1990-2020 (1990-2017)	20 (1)	2001-2020 (2011-2011)
**Bangladesh**	31 (13)	1990-2020 (1990-2018)	28 (2)	1993-2020 (2015-2018)
**Bulgaria**	31 (6)	1990-2020 (1990-2015)	19 (0)	2002-2020 (-)
**Bahrain**	31 (6)	1990-2020 (1990-2015)	0 (0)	- (-)
**Bahamas, The**	31 (6)	1990-2020 (1990-2015)	0 (0)	- (-)
**Bosnia and Herzegovina**	31 (6)	1990-2020 (1995-2015)	13 (0)	2008-2020 (-)
**Belarus**	31 (8)	1990-2020 (1990-2015)	13 (0)	2008-2020 (-)
**Belize**	31 (6)	1990-2020 (1990-2015)	13 (0)	2008-2020 (-)
**Bolivia**	31 (11)	1990-2020 (1990-2017)	23 (1)	1998-2020 (2016-2016)
**Brazil**	31 (13)	1990-2020 (1990-2018)	28 (2)	1993-2020 (2003-2018)
**Barbados**	31 (6)	1990-2020 (1990-2015)	0 (0)	- (-)
**Brunei Darussalam**	31 (6)	1990-2020 (1990-2015)	0 (0)	- (-)
**Bhutan**	31 (6)	1990-2020 (1990-2015)	14 (0)	2007-2020 (-)
**Botswana**	31 (9)	1990-2020 (1990-2015)	13 (0)	2008-2020 (-)
**Central African Republic**	31 (8)	1990-2020 (1990-2016)	25 (0)	1996-2020 (-)
**Canada**	31 (7)	1990-2020 (1990-2015)	31 (0)	1990-2020 (-)
**Switzerland**	31 (6)	1990-2020 (1990-2015)	28 (0)	1993-2020 (-)
**Chile**	31 (11)	1990-2020 (1990-2018)	13 (1)	2008-2020 (2018-2018)
**China**	31 (8)	1990-2020 (1990-2018)	18 (2)	2003-2020 (2013-2018)
**Côte d'Ivoire**	31 (9)	1990-2020 (1990-2016)	19 (0)	2002-2020 (-)
**Cameroon**	31 (10)	1990-2020 (1990-2015)	27 (2)	1994-2020 (2011-2014)
**Democratic Republic of the Congo**	31 (9)	1990-2020 (1990-2015)	24 (1)	1997-2020 (2014-2014)
**Congo, Rep.**	31 (7)	1990-2020 (1990-2015)	0 (0)	- (-)
**Colombia**	31 (10)	1990-2020 (1990-2018)	26 (3)	1995-2020 (2005-2018)
**Comoros**	31 (6)	1990-2020 (1990-2015)	19 (0)	2002-2020 (-)
**Cape Verde**	31 (6)	1990-2020 (1990-2015)	26 (1)	1995-2020 (2005-2005)
**Costa Rica**	31 (10)	1990-2020 (1990-2018)	28 (1)	1993-2020 (2018-2018)
**Cuba**	29 (5)	1992-2020 (1995-2015)	13 (0)	2008-2020 (-)
**Cyprus**	31 (8)	1990-2020 (1990-2015)	19 (0)	2002-2020 (-)
**Czech Republic**	31 (5)	1990-2020 (1995-2015)	28 (0)	1993-2020 (-)
**Germany**	31 (7)	1990-2020 (1990-2015)	19 (0)	2002-2020 (-)
**Djibouti**	31 (6)	1990-2020 (1990-2015)	0 (0)	- (-)
**Dominica**	10 (1)	2011-2020 (2015-2015)	0 (0)	- (-)
**Denmark**	31 (6)	1990-2020 (1990-2015)	28 (0)	1993-2020 (-)
**Dominican Republic**	31 (11)	1990-2020 (1990-2018)	29 (2)	1992-2020 (2013-2018)
**Algeria**	31 (10)	1990-2020 (1990-2015)	0 (0)	- (-)
**Ecuador**	31 (9)	1990-2020 (1990-2018)	27 (1)	1994-2020 (2018-2018)
**Egypt**	31 (11)	1990-2020 (1990-2018)	26 (3)	1995-2020 (2005-2018)
**Eritrea**	31 (5)	1990-2020 (1995-2015)	0 (0)	- (-)
**Spain**	31 (7)	1990-2020 (1990-2015)	19 (0)	2002-2020 (-)
**Estonia**	31 (7)	1990-2020 (1990-2015)	19 (0)	2002-2020 (-)
**Ethiopia**	31 (10)	1990-2020 (1990-2016)	28 (1)	1993-2020 (2016-2016)
**Finland**	31 (6)	1990-2020 (1990-2015)	19 (0)	2002-2020 (-)
**Fiji**	31 (6)	1990-2020 (1990-2015)	20 (0)	2001-2020 (-)
**France**	31 (6)	1990-2020 (1990-2015)	31 (1)	1990-2020 (2000-2000)
**Micronesia (Federated States of)**	31 (6)	1990-2020 (1990-2015)	17 (0)	2004-2020 (-)
**Gabon**	31 (9)	1990-2020 (1990-2017)	19 (1)	2002-2020 (2012-2012)
**United Kingdom**	31 (6)	1990-2020 (1990-2015)	26 (1)	1995-2020 (2005-2005)
**Georgia**	31 (10)	1990-2020 (1990-2018)	26 (2)	1995-2020 (2005-2018)
**Ghana**	31 (14)	1990-2020 (1990-2017)	23 (1)	1998-2020 (2008-2008)
**Guinea**	31 (7)	1990-2020 (1990-2015)	13 (0)	2008-2020 (-)
**Gambia**	31 (8)	1990-2020 (1990-2015)	18 (1)	2003-2020 (2013-2013)
**Guinea-Bissau**	31 (7)	1990-2020 (1990-2015)	0 (0)	- (-)
**Equatorial Guinea**	31 (6)	1990-2020 (1990-2015)	20 (0)	2001-2020 (-)

**Greece**	31 (7)	1990-2020 (1990-2017)	19 (0)	2002-2020 (-)
**Grenada**	31 (6)	1990-2020 (1990-2015)	13 (0)	2008-2020 (-)
**Guatemala**	31 (7)	1990-2020 (1990-2015)	22 (2)	1999-2020 (2009-2015)
**Guyana**	31 (6)	1990-2020 (1990-2015)	13 (0)	2008-2020 (-)
**Hong Kong SAR, China**	7 (0)	2014-2020 (-)	7 (0)	2014-2020 (-)
**Honduras**	31 (9)	1990-2020 (1990-2018)	25 (2)	1996-2020 (2012-2018)
**Croatia**	31 (5)	1990-2020 (1995-2015)	19 (0)	2002-2020 (-)
**Haiti**	31 (7)	1990-2020 (1990-2016)	31 (1)	1990-2020 (2000-2000)
**Hungary**	31 (6)	1990-2020 (1990-2015)	19 (0)	2002-2020 (-)
**Indonesia**	31 (12)	1990-2020 (1990-2018)	13 (1)	2008-2020 (2018-2018)
**India**	31 (11)	1990-2020 (1990-2017)	25 (2)	1996-2020 (2006-2016)
**Ireland**	31 (6)	1990-2020 (1990-2015)	19 (0)	2002-2020 (-)
**Iran (Islamic Republic of)**	31 (6)	1990-2020 (1990-2015)	13 (0)	2008-2020 (-)
**Iraq**	31 (8)	1990-2020 (1990-2018)	0 (0)	- (-)
**Iceland**	31 (6)	1990-2020 (1990-2015)	23 (0)	1998-2020 (-)
**Israel**	31 (7)	1990-2020 (1990-2016)	13 (0)	2008-2020 (-)
**Italy**	31 (6)	1990-2020 (1990-2015)	25 (0)	1996-2020 (-)
**Jamaica**	31 (7)	1990-2020 (1990-2015)	22 (1)	1999-2020 (2009-2009)
**Jordan**	31 (10)	1990-2020 (1990-2018)	24 (2)	1997-2020 (2012-2018)
**Japan**	31 (6)	1990-2020 (1990-2015)	28 (0)	1993-2020 (-)
**Kazakhstan**	31 (10)	1990-2020 (1990-2018)	20 (3)	2001-2020 (2011-2018)
**Kenya**	31 (13)	1990-2020 (1990-2017)	28 (2)	1993-2020 (2009-2014)
**Kyrgyz Republic**	31 (9)	1990-2020 (1990-2018)	19 (2)	2002-2020 (2012-2018)
**Cambodia**	31 (8)	1990-2020 (1995-2015)	31 (4)	1990-2020 (2000-2014)
**Kiribati**	31 (6)	1990-2020 (1990-2015)	23 (0)	1998-2020 (-)
**South Korea**	31 (7)	1990-2020 (1990-2018)	13 (1)	2008-2020 (2018-2018)
**Kuwait**	31 (5)	1990-2020 (1995-2015)	0 (0)	- (-)
**Lao People's Democratic Republic**	31 (7)	1990-2020 (1990-2017)	17 (0)	2004-2020 (-)
**Lebanon**	31 (8)	1990-2020 (1990-2018)	0 (0)	- (-)
**Liberia**	29 (7)	1992-2020 (1995-2015)	24 (1)	1997-2020 (2007-2007)
**Libya**	31 (7)	1990-2020 (1995-2015)	0 (0)	- (-)
**St. Lucia**	31 (6)	1990-2020 (1990-2015)	0 (0)	- (-)
**Sri Lanka**	31 (12)	1990-2020 (1990-2017)	18 (1)	2003-2020 (2013-2013)
**Lesotho**	31 (11)	1990-2020 (1990-2018)	13 (1)	2008-2020 (2018-2018)
**Lithuania**	31 (6)	1990-2020 (1990-2015)	19 (0)	2002-2020 (-)
**Luxembourg**	31 (6)	1990-2020 (1990-2015)	19 (0)	2002-2020 (-)
**Latvia**	31 (5)	1990-2020 (1995-2015)	19 (0)	2002-2020 (-)
**Morocco**	31 (11)	1990-2020 (1990-2015)	21 (1)	2000-2020 (2010-2010)
**Republic of Moldova**	31 (8)	1990-2020 (1990-2015)	26 (1)	1995-2020 (2005-2005)
**Madagascar**	31 (7)	1990-2020 (1990-2015)	13 (0)	2008-2020 (-)
**Maldives**	31 (6)	1990-2020 (1990-2015)	25 (0)	1996-2020 (-)
**Mexico**	31 (8)	1990-2020 (1990-2018)	15 (1)	2006-2020 (2018-2018)
**Marshall Islands**	13 (0)	2008-2020 (-)	13 (0)	2008-2020 (-)
**North Macedonia**	31 (5)	1990-2020 (1995-2015)	13 (0)	2008-2020 (-)
**Mali**	31 (10)	1990-2020 (1990-2018)	25 (1)	1996-2020 (2018-2018)
**Malta**	31 (6)	1990-2020 (1990-2015)	21 (1)	2000-2020 (2010-2010)
**Myanmar**	31 (12)	1990-2020 (1990-2017)	15 (1)	2006-2020 (2016-2016)
**Montenegro**	21 (4)	2000-2020 (2005-2018)	13 (1)	2008-2020 (2018-2018)
**Mongolia**	31 (6)	1990-2020 (1995-2018)	14 (1)	2007-2020 (2018-2018)
**Mozambique**	31 (10)	1990-2020 (1990-2015)	27 (2)	1994-2020 (2011-2015)
**Mauritania**	31 (6)	1990-2020 (1990-2015)	0 (0)	- (-)
**Mauritius**	31 (6)	1990-2020 (1990-2015)	0 (0)	- (-)
**Malawi**	31 (11)	1990-2020 (1990-2016)	27 (3)	1994-2020 (2004-2016)
**Malaysia**	31 (10)	1990-2020 (1990-2018)	0 (0)	- (-)
**Namibia**	31 (11)	1990-2020 (1990-2015)	28 (2)	1993-2020 (2003-2013)
**Niger**	31 (10)	1990-2020 (1990-2015)	13 (0)	2008-2020 (-)
**Nigeria**	31 (10)	1990-2020 (1990-2018)	23 (3)	1998-2020 (2008-2018)
**Nicaragua**	31 (6)	1990-2020 (1990-2015)	24 (0)	1997-2020 (-)
**Netherlands**	31 (7)	1990-2020 (1990-2015)	19 (1)	2002-2020 (2012-2012)
**Norway**	31 (6)	1990-2020 (1990-2015)	23 (0)	1998-2020 (-)
**Nepal**	31 (9)	1990-2020 (1990-2016)	20 (2)	2001-2020 (2011-2016)
**New Zealand**	31 (7)	1990-2020 (1990-2015)	29 (0)	1992-2020 (-)
**Oman**	31 (5)	1990-2020 (1995-2015)	0 (0)	- (-)
**Pakistan**	31 (13)	1990-2020 (1990-2018)	13 (1)	2008-2020 (2018-2018)
**Panama**	31 (8)	1990-2020 (1990-2015)	22 (0)	1999-2020 (-)
**Peru**	31 (13)	1990-2020 (1990-2018)	28 (9)	1993-2020 (2010-2018)
**Philippines**	31 (9)	1990-2020 (1990-2017)	26 (2)	1995-2020 (2005-2017)
**Papua New Guinea**	31 (6)	1990-2020 (1990-2015)	18 (0)	2003-2020 (-)
**Poland**	31 (7)	1990-2020 (1990-2015)	27 (1)	1994-2020 (2012-2012)
**Puerto Rico**	31 (6)	1990-2020 (1990-2015)	0 (0)	- (-)
**Korea, Dem. People's Rep.**	11 (0)	2010-2020 (-)	0 (0)	- (-)
**Portugal**	31 (6)	1990-2020 (1990-2015)	19 (0)	2002-2020 (-)
**Paraguay**	31 (10)	1990-2020 (1990-2018)	23 (2)	1998-2020 (2008-2018)
**Occupied Palestinian territory**	31 (5)	1990-2020 (1995-2015)	13 (0)	2008-2020 (-)
**Qatar**	31 (7)	1990-2020 (1990-2015)	0 (0)	- (-)

**Romania**	31 (8)	1990-2020 (1990-2018)	19 (2)	2002-2020 (2012-2018)
**Russia**	31 (8)	1990-2020 (1990-2017)	0 (0)	- (-)
**Rwanda**	31 (12)	1990-2020 (1990-2016)	26 (3)	1995-2020 (2005-2015)
**Saudi Arabia**	31 (5)	1990-2020 (1995-2015)	0 (0)	- (-)
**Sudan**	31 (10)	1990-2020 (1990-2015)	13 (0)	2008-2020 (-)
**Senegal**	31 (12)	1990-2020 (1990-2017)	14 (1)	2007-2020 (2017-2017)
**Singapore**	31 (7)	1990-2020 (1990-2015)	22 (0)	1999-2020 (-)
**Solomon Islands**	31 (6)	1990-2020 (1990-2015)	23 (0)	1998-2020 (-)
**Sierra Leone**	31 (10)	1990-2020 (1990-2017)	18 (1)	2003-2020 (2013-2013)
**El Salvador**	31 (12)	1990-2020 (1990-2018)	23 (3)	1998-2020 (2008-2018)
**Somalia**	31 (6)	1990-2020 (1990-2015)	0 (0)	- (-)
**Serbia**	31 (6)	1990-2020 (1995-2017)	28 (0)	1993-2020 (-)
**South Sudan**	13 (2)	2008-2020 (2010-2015)	13 (0)	2008-2020 (-)
**São Tomé and Principe**	31 (6)	1990-2020 (1990-2015)	22 (0)	1999-2020 (-)
**Suriname**	31 (6)	1990-2020 (1990-2015)	13 (0)	2008-2020 (-)
**Slovak Republic**	31 (6)	1990-2020 (1990-2015)	23 (0)	1998-2020 (-)
**Slovenia**	31 (7)	1990-2020 (1990-2015)	19 (0)	2002-2020 (-)
**Sweden**	31 (7)	1990-2020 (1990-2015)	19 (0)	2002-2020 (-)
**Eswatini**	31 (10)	1990-2020 (1990-2016)	13 (0)	2008-2020 (-)
**Seychelles**	31 (6)	1990-2020 (1990-2015)	0 (0)	- (-)
**Syrian Arab Republic**	31 (6)	1990-2020 (1990-2015)	0 (0)	- (-)
**Chad**	31 (6)	1990-2020 (1990-2015)	16 (1)	2005-2020 (2015-2015)
**Togo**	31 (8)	1990-2020 (1990-2017)	17 (0)	2004-2020 (-)
**Thailand**	31 (14)	1990-2020 (1990-2018)	28 (1)	1993-2020 (2018-2018)
**Tajikistan**	31 (6)	1990-2020 (1990-2015)	19 (0)	2002-2020 (-)
**Turkmenistan**	31 (5)	1990-2020 (1995-2015)	0 (0)	- (-)
**Timor-Leste**	31 (5)	1990-2020 (1995-2015)	21 (1)	2000-2020 (2010-2010)
**Tonga**	31 (7)	1990-2020 (1990-2015)	22 (0)	1999-2020 (-)
**Trinidad and Tobago**	31 (6)	1990-2020 (1990-2015)	14 (0)	2007-2020 (-)
**Tunisia**	31 (9)	1990-2020 (1990-2018)	13 (1)	2008-2020 (2018-2018)
**Turkey**	31 (9)	1990-2020 (1990-2018)	23 (1)	1998-2020 (2018-2018)
**Taiwan, China**	16 (0)	2005-2020 (-)	0 (0)	- (-)
**United Republic of Tanzania**	31 (13)	1990-2020 (1990-2017)	28 (3)	1993-2020 (2003-2016)
**Uganda**	31 (13)	1990-2020 (1990-2017)	25 (3)	1996-2020 (2006-2016)
**Ukraine**	31 (9)	1990-2020 (1990-2015)	24 (0)	1997-2020 (-)
**Uruguay**	31 (10)	1990-2020 (1990-2018)	18 (2)	2003-2020 (2013-2018)
**United States of America**	31 (9)	1990-2020 (1990-2017)	31 (1)	1990-2020 (2000-2000)
**Uzbekistan**	31 (6)	1990-2020 (1990-2015)	0 (0)	- (-)
**St. Vincent and the Grenadines**	31 (6)	1990-2020 (1990-2015)	0 (0)	- (-)
**Venezuela**	31 (6)	1990-2020 (1990-2015)	13 (0)	2008-2020 (-)
**Virgin Islands (U.S.)**	3 (0)	2018-2020 (-)	0 (0)	- (-)
**Vietnam**	31 (11)	1990-2020 (1990-2016)	21 (1)	2000-2020 (2010-2010)
**Vanuatu**	31 (6)	1990-2020 (1990-2015)	22 (0)	1999-2020 (-)
**Samoa**	31 (6)	1990-2020 (1990-2015)	28 (0)	1993-2020 (-)
**Yemen, Rep.**	31 (6)	1990-2020 (1990-2015)	0 (0)	- (-)
**South Africa**	31 (14)	1990-2020 (1990-2017)	15 (1)	2006-2020 (2016-2016)
**Zambia**	31 (11)	1990-2020 (1990-2018)	24 (3)	1997-2020 (2007-2018)
**Zimbabwe**	31 (11)	1990-2020 (1990-2017)	25 (3)	1996-2020 (2006-2015)

Appendix D. Example Stata gsem syntax

```
gsem (conf25 i_natdis_sev5 colever i_unwntnei_high2 <-, logit) ///
(i_lngdp  i_gini i_lnhomi <- ) ///
(i_gii <-, family(beta) link(logit)) ///
(C <- c.yr), ///
lclass (C 3) startvalues(randompr, draws(200) seed (10)) ///
vce(cluster geocode) ///
covstructure(e._OEn, unstructured) ///
lcinvariant(none) ///
nodvheader
```

NB: i_ prefix indicates variable is partially imputed (i.e. we forward filled gaps in estimates with existing data).

Appendix E. Table A4: LCA Goodness of Fit and Class Separation Statistics

							LMR LT (k vs k-1)		Class 1				Class 2				Class 3				Class 4				Class 5			
**k**	**LL**	**df**	**AIC**	**BIC**	**SSBIC**	**E**	**Test stat.**	**P**	**n**	**AvePP**	**Prop.**	**OCC**	**n**	**AvePP**	**Prop.**	**OCC**	**n**	**AvePP**	**Prop.**	**OCC**	**n**	**AvePP**	**Prop.**	**OCC**	**n**	**AvePP**	**Prop.**	**OCC**
1	-43105	15	86240	86340	86303	.	.	.		1.00	1.00	.																
2	-40157	32	80378	80591	80501	**0.88**	5677	<0.001	4,327	**0.98**	0.75	13.10	1,405	**0.94**	0.25	43.81												
3	-39070	49	78238	78564	78419	0.77	2094	<0.001	1,386	0.93	0.24	43.58	2,262	0.90	0.40	13.89	2,084	0.86	0.36	10.78								
4	-38593	64	77314	77740	77558	0.78	919	<0.001	2,214	0.90	0.38	14.60	1,361	0.87	0.23	22.94	1,177	**0.91**	0.20	39.00	980	0.81	0.18	18.71				
5	-38286	81	**76735**	**77274**	**77038**	0.79	591	<0.001	1,365	0.87	0.24	22.08	753	0.84	0.13	35.41	2,253	0.90	0.39	13.95	882	0.81	0.16	21.56	479	0.88	0.08	80.99

N = 5,732 country-years. K: number of classes in latent class model. Lower AIC (Akaike Information Criterion) and BIC (Bayesian Information Criterion) values indicate better model fit (SSBIC: Sample size adjusted BIC) ; the lowest values are highlighted in bold. Entropy values approaching 1 indicate clear delineation of classes, whilst entropy values less than 0.8 are problematic if most likely class membership is used as a variable in further analysis; entropy values less than 0.8 are highlighted in yellow, and highest entropy value highlighted in bold. LMR LT: Lo-Mendell-Rubin Likelihood Ratio Test of k − 1 classes against k classes, where a significant result thereby indicates that the null hypothesis of k − 1 classes should be rejected in favour of at least k classes. AvePP (Average Posterior Probability) should be at least 0.7 for all classes and is so in all models, and highest AvePPs are highlighted in bold. Larger values of Odds of Correct Classification (OCC) indicate better assignment accuracy and an OCC greater than 5 for all classes is indicative that the model has high assignment accuracy (the case here for all models), highest OCCs shown in bold. Models adjusted for the effect of time and clustering at the country level. Models with 1-5 classes all converged successfully. However, the 4 and 5 class models needed some parameters constrained as probabilities were very close to 0 or 1 (conflict was constrained to 15 in Class 2 in the 5 class model and Class 1 in the 4 class model and social discrimination to -15 in Class 3 in the 5 class model and Class 2 in the 4 class model) (Ng, 2018b; Ng & Schechter, 2017).

Appendix F. Data acknowledgements

Many thanks to the various statistical agencies from around the world involved in producing the original data, including those listed in the table below:

Argentina	National Institute of Statistics and Censuses
Armenia	National Statistical Service
Austria	National Bureau of Statistics
Bangladesh	Bureau of Statistics
Belarus	Ministry of Statistics and Analysis
Benin	National Institute of Statistics and Economic Analysis
Bolivia	National Institute of Statistics
Botswana	Central Statistics Office
Brazil	Institute of Geography and Statistics
Burkina Faso	National Institute of Statistics and Demography
Cambodia	National Institute of Statistics
Cameroon	Central Bureau of Census and Population Studies
Canada	Statistics Canada
Chile	National Institute of Statistics
China	National Bureau of Statistics
Colombia	National Administrative Department of Statistics
Costa Rica	National Institute of Statistics and Censuses
Cuba	Office of National Statistics
Dominican Republic	National Statistics Office
Ecuador	National Institute of Statistics and Censuses
Egypt	Central Agency for Public Mobilization and Statistics
El Salvador	General Directorate of Statistics and Censuses
Ethiopia	Central Statistical Agency
Fiji Islands	Bureau of Statistics
France	National Institute of Statistics and Economic Studies
Germany	Federal Statistical Office
Ghana	Ghana Statistical Services
Greece	National Statistical Office
Guatemala	National Institute of Statistics
Guinea	National Statistics Directorate
Haiti	Institute of Statistics and Informatics
Honduras	National Institute of Statistics
Hungary	Central Statistical Office
India	Ministry of Statistics and Programme Implementation
Indonesia	Statistics Indonesia
Iraq	Central Statistical Office
Ireland	Central Statistics Office
Israel	Central Bureau of Statistics
Italy	National Institute of Statistics
Jamaica	Statistical Institute
Jordan	Department of Statistics
Kenya	National Bureau of Statistics
Kyrgyz Republic	National Statistical Committee
Laos	Statistics Bureau
Lesotho	Bureau of Statistics
Liberia	Institute of Statistics and Geo-Information Systems
Malawi	National Statistical Office
Malaysia	Department of Statistics

Mali	National Directorate of Statistics and Informatics
Mexico	National Institute of Statistics, Geography, and Informatics
Mongolia	National Statistical Office
Morocco	High Commission of Planning
Mozambique	National Institute of Statistics
Nepal	Central Bureau of Statistics
Netherlands	Statistics Netherlands
Nicaragua	National Institute of Statistics and Censuses
Nigeria	National Bureau of Statistics
Pakistan	Statistics Division
Palestine	Central Bureau of Statistics
Panama	Census and Statistics Directorate
Papua New Guinea	National Statistical Office
Paraguay	General Directorate of Statistics, Surveys, and Censuses
Peru	National Institute of Statistics and Informatics
Philippines	National Statistics Office
Poland	Central Statistical Office
Portugal	National Institute of Statistics
Puerto Rico	U.S. Bureau of the Census
